# Progress to a Gallium-Arsenide Deep-Center Laser

**DOI:** 10.3390/ma2041599

**Published:** 2009-10-22

**Authors:** Janet L. Pan

**Affiliations:** Yale University, P.O. Box 208284, New Haven, CT 06520-8284, USA; E-Mail: janet.pan@yale.edu; Tel.: +1-203-432-4733; Fax: +1-203-432-6420

**Keywords:** laser action, stimulated emission, electroluminescence, photoluminescence, gallium-arsenide, deep-centers

## Abstract

Although photoluminescence from gallium-arsenide (GaAs) deep-centers was first observed in the 1960s, semiconductor lasers have always utilized conduction-to-valence-band transitions. Here we review recent materials studies leading to the first GaAs deep-center laser. First, we summarize well-known properties: nature of deep-center complexes, Franck-Condon effect, photoluminescence. Second, we describe our recent work: insensitivity of photoluminescence with heating, striking differences between electroluminescence and photoluminescence, correlation between transitions to deep-states and absence of bandgap-emission. Room-temperature stimulated-emission from GaAs deep-centers was observed at low electrical injection, and could be tuned from the bandgap to half-the-bandgap (900–1,600 nm) by changing the electrical injection. The first GaAs deep-center laser was demonstrated with electrical injection, and exhibited a threshold of less than 27 mA/cm${}^{2}$ in continuous-wave mode at room temperature at the important 1.54 *μ*m fiber-optic wavelength. This small injection for laser action was explained by fast depopulation of the lower state of the optical transition (fast capture of free holes onto deep-centers), which maintains the population inversion. The evidence for laser action included: superlinear L-I curve, quasi-Fermi level separations satisfying Bernard-Duraffourg’s criterion, optical gains larger than known significant losses, clamping of the optical-emission from lossy modes unable to reach laser action, pinning of the population distribution during laser action.

## 1. Introduction

The ongoing quest for “thresholdless” [1] semiconductor lasers has led to the development of new materials (e.g., quantum wells [2], wires, and dots [3,4]) and new optical resonators (e.g., microdisks [4] and photonic bandgap crystals [1]). In a novel effort towards “thresholdless” lasers, we recently demonstrated [5] that native deep-acceptor complexes in gallium-arsenide (GaAs) exhibited laser action at very low current densities. Moreover, in contrast to conventional semiconductor devices, whose operating wavelengths are determined by the bandgap energy, we showed [6,7] that the room-temperature stimulated-emission from GaAs deep-centers can be tuned very widely from the bandgap (∼900 nm) to half-the-bandgap (1600 nm). Here we review both historical work and our progress towards this first GaAs deep-center laser.

First, in Section 3, we summarize some well-known properties of deep-centers in highly n-doped GaAs: the nature of the deep-acceptor complexes, the Franck-Condon effect, the observed photoluminescence. Second, we describe our recent work on GaAs deep-centers: the total radiative output in photoluminescence, the insensitivity of the photoluminescence with respect to a 90 ${}^{\circ }$C rise above room temperature, the dependence of the photoluminescence (PL) and electroluminescence (EL) on the pump power, a correlation between transitions to deep-states and the absence of bandgap emission, the fast capture of free holes onto deep-centers. An important aspect of our work was the observation of a significant difference between photoluminescence and electroluminescence. In our work, the PL could not be saturated, and the PL spectra retain the same shape for all optical pump powers. In stark contrast, the EL is found to saturate at long wavelengths, and to show a strong spectral blue-shift with increasing injection. These observations were explained by a small hole diffusion length and fast capture of free holes onto deep-centers. This fast capture of free holes onto deep-centers is consistent with the absence from all our deep-center samples of bandedge emission in photoluminescence.

Next, in Section 5, we report our work on room-temperature stimulated-emission from GaAs deep-centers at low electrical injection. The evidence for stimulated-emission includes: a superlinear L-I curve, a quasi-Fermi level separation large enough to satisfy the Bernard-Duraffourg criterion, and an optical gain large enough to overcome significant loss. We found that the room-temperature stimulated-emission from GaAs deep-centers can be tuned very widely from the bandgap (about 900 nm) to half-the-bandgap (1600 nm) by changing the electrical injection.

Section 6 presents our work on the GaAs deep-center laser. The first GaAs deep-center laser was demonstrated with electrical injection, and exhibited a threshold of less than 27 mA/cm${}^{2}$ in continuous-wave mode at room temperature at the important 1.54 *μ*m fiber-optic wavelength. This small injection which achieves laser action can be explained by a fast depopulation of the lower state of the optical transition (i.e., fast capture of free holes onto deep-centers). The latter helps to maintain the population inversion. The evidence for laser action included: a superlinear L-I curve, an optical gain large enough to overcome significant loss, a clamping of the optical emission from lossy modes that do not show laser action, and a pinning of the population distribution during laser action.

## 2. Methods

In our work, all samples were grown by molecular beam epitaxy on semi-insulating GaAs substrates. All samples were representative of several dozen growths. We chose to write about these specific samples because we had taken more comprehensive data from these samples. Sample A consists of 2,488 Å of the GaAs deep-centers, above which was a 1,418 Å GaAs layer p-doped at 3.2 × 10${}^{19}$ cm${}^{-3}$. Sample B consists of 2,974 Å of the GaAs deep-centers above which was no p-layer. Sample C consists of 2,271 Å of the GaAs deep-centers, above which was a 1,294 Å GaAs layer p-doped at 2.5 × 10${}^{19}$ cm${}^{-3}$. The GaAs deep-center layers in samples A, B, C were grown at 570 ${}^{\circ }$C under As-rich conditions and high Si-dopant flux (4.5 × 10${}^{19}$ cm${}^{-3}$). Sample D was a control sample of 21 periods of high-quality 120 Å InGaAs/120 Å InAlAs MQWs lattice-matched to indium phosphide (InP). Sample E was a control sample of 2,325 Å of bulk InGaAs lattice-matched to InP. Transmission, photoluminescence (PL), and Hall measurements were performed on samples A, B and C. Electroluminescence (EL) and current-voltage were measured on samples A and C. PL was performed on samples D and E.

Sample F consisted of 3000 Å of undoped GaAs buffer, 1,149 Å of AlAs etch stop, 2,271 Å of GaAs deep-centers, 399 Å of Al${}_{0.45}$Ga${}_{0.55}$As, above which was a 1,294 Å GaAs layer p-doped at 3.2 × 10${}^{19}$ cm${}^{-3}$. Sample G consisted of 3,000 Å of undoped GaAs buffer, 42 periods of a distributed Bragg reflector (DBR, 1,148 Å of GaAs and 1,340 Å of AlAs), 2,486 Å of GaAs deep-centers, 399 Å of Al${}_{0.45}$Ga${}_{0.55}$As, above which was a 3143 Å GaAs layer p-doped at 3.2 × 10${}^{19}$ cm${}^{-3}$. The growth which yielded Samples H-N consisted of 3,000 Å of undoped GaAs buffer, 35 periods of a DBR (1104 Å of GaAs and 1,266 Å of Al${}_{0.86}$Ga${}_{0.14}$As), 2,108 Å of GaAs deep-centers, 399 Å of Al${}_{0.45}$Ga${}_{0.55}$As, above which was a 1,937 Å GaAs layer p-doped at 3.2 × 10${}^{19}$ cm${}^{-3}$. The Si donor concentration in the deep-center layer was always 4.5-4.8 × 10${}^{19}$ cm${}^{-3}$. We estimate that the concentration of Si${}_{\text{Ga}}$-V${}_{\text{Ga}}$ in the deep-center layer was about ∼1.5-2 × 10${}^{19}$ cm${}^{-3}$. Further details were reported previously [7].

**Figure 1:**
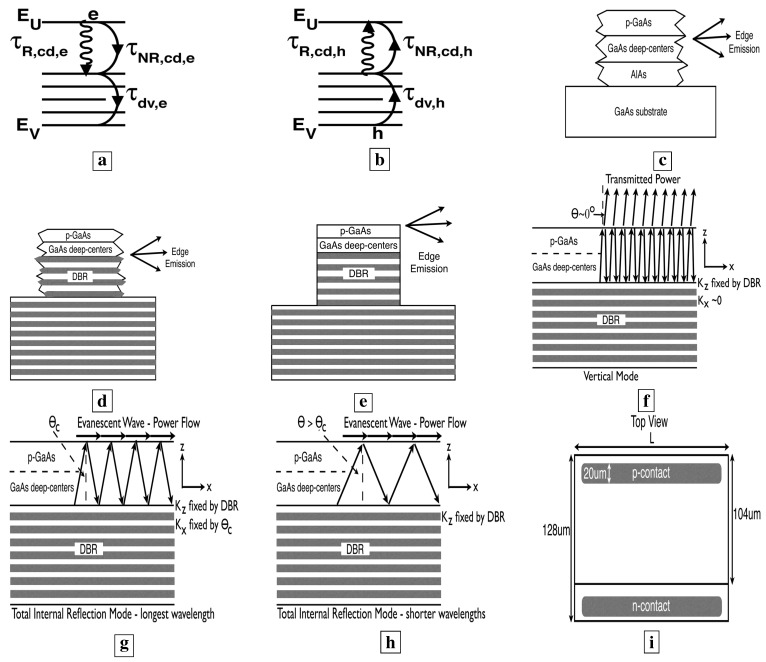
Lifetimes and measurement geometries [5]. **a,** Lifetimes for electrons. **b,** Lifetimes for holes. **c,** Single-pass measurement from the edge of Sample F. The active layer was *not* inside a resonant cavity or waveguide. **d,** Single-pass measurement from the edge of Sample G. A bottom mirror (DBR) was added to increase the single-pass optical length. The rough wet-etched facets preclude the optical-feedback characteristic of resonant cavities. **e,** Edge emission from Samples H-N. The RIE facets allow a resonant cavity, with its characteristic optical-feedback. **f,** Lossy “vertical” waveguide mode, which is normally incident upon the sample surface and whose longitudinal wave vector K${}_{\text{X}}$ is nearly zero. 70% of the incident power is transmitted vertically and lost through the top surface. **g,** Low-loss longest wavelength total-internal-reflection (TIR) mode. Here, rays from within the semiconductor are incident upon the sample surface at the critical angle $\theta_{\text{C}}$ for TIR. **h,** Shorter wavelength TIR mode. Here, rays from within the semiconductor are incident upon the sample surface at an angle greater than $\theta_{\text{C}}$. This mode makes fewer passes through the active region. **i,** Top view of the semiconductor surface showing the pixel dimensions.

Devices were fabricated using standard photolithography, wet etches, and Ti-Au contacts. Pixels are shown in Figure 1i. Individual devices were isolated from each other by etching 128 *μ*m × L mesas in the n-type deep-center layer. Current was injected through 104 *μ*m × L mesas in the p-type GaAs layer. In Samples F and G, L was 75 *μ*m and 150 *μ*m, respectively. The pixels in Samples F and G were fabricated with wet etches (phosphoric acid). The wet etches left the mesa edges with a random roughness. The isolation etch of Sample G extended 3.5 periods into the DBR. The pixels in Samples H-N were fabricated via reactive ion etching (RIE) with an inductively coupled plasma. The isolation etch of Samples H-N extended 4.5 periods into the DBR. The Ti-Au contacts were 20 *μ*m wide. The electrical injection utilized current pulses which were 25-400 *μ*s wide at 50% duty cycle. The optical emission was measured through a SPEX 1681B spectrometer, and collected by either an InGaAs photodetector or a photomultiplier tube.

All samples had similar layer structures, and were operated at similar current densities. The main difference between the samples F-N was the presence or absence of a resonant cavity. Sample F was *not* placed within a resonant cavity or waveguide. A DBR was placed underneath the active layer in Samples G-N to increase the optical path for resonant normal wave vectors K${}_{\text{Z}}$. In Samples F and G, Figures 1c-d respectively show that wet-etched rough facets preclude the optical-feedback characteristic of resonant cavities. In the six Samples H-N, Figure 1e shows that RIE facets made possible a resonant cavity and optical-feedback.

## 3. Photoluminescence and Electroluminescence Studies

**Figure 2:**
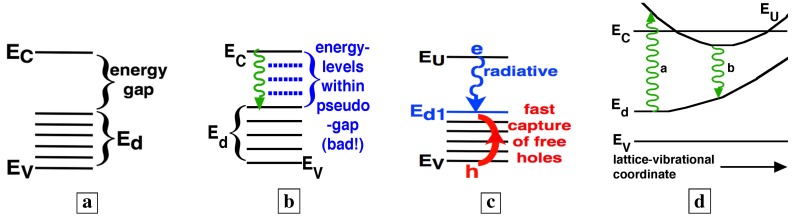
**a,** Energies [5,6,7] associated with deep-centers in GaAs. **b,** The dashed lines indicate energies which lie between the upper-state (near the conduction-band) and lower-state (near midgap) of an optical transition. **c,** Radiative transition between a state E${}_{\text{U}}$ near the conduction-band and a deep-state E${}_{\text{d1}}$. Free holes undergo fast capture onto deep-centers. **d,** Energy diagram showing both the coupling to the lattice-vibrational-coordinate, and the Franck-Condon shift of absorption to higher energies than the emission.

We recently [7] developed a new growth technique which uses a large n-type doping to thermodynamically favor the formation of large concentrations of compensating deep-acceptors. The deep-acceptors effectively compensate the material only if their associated energy-levels lie below the midgap. (Deep-levels which lie above the midgap are usually donor levels.) Our growth conditions thus favor the formation of deep-levels below midgap, and not above midgap. This allows the formation of a high-quality pseudo-bandgap between the conduction-band and midgap (Figure 2a). The relative absence of states within this pseudo-bandgap makes the radiative efficiency large. Thus, the new material has energies as in Figure 2a, rather than Figure 2b. In Figure 2b-c, E${}_{\text{C}}$, E${}_{\text{V}}$, E${}_{\text{d}}$, and E${}_{\text{U}}$ are, respectively, the conduction- and valence-band edges, the deep-levels, and an upper-state resonant with the conduction-band. Figure 2c shows the radiative transition between the state E${}_{\text{U}}$ near the conduction-band and a deep-state E${}_{\text{d1}}$, as well as the fast capture [7] of free holes onto deep-centers. The literature [8,9,10] says that the upper state E${}_{\text{U}}$ corresponds to a state centered on the donor in a donor-V${}_{\text{Ga}}$ complex, whereas the lower state E${}_{\text{d}}$ corresponds to a state centered on the V${}_{\text{Ga}}$ in the complex.

Though these deep-acceptors have been known since the 1960s [8,11,12,13,14,15,16,17,18], this material is novel because n-type GaAs is not usually designed to retain large concentrations of compensating acceptors. These compensating acceptors are [8,11,19,20,21,22,23] donor-vacancy-on-gallium complexes (donor-V${}_{\text{Ga}}$ complexes, e.g., V${}_{\text{Ga}}$, Si${}_{\text{Ga}}$-V${}_{\text{Ga}}$, Si${}_{\text{Ga}}$-V${}_{\text{Ga}}$-Si${}_{\text{Ga}}$, etc., where Si${}_{\text{Ga}}$ denotes the silicon-on-gallium-site). These donor-V${}_{\text{Ga}}$ complexes are known [8,11,13,17,18] to show a Franck-Condon spectral shift of absorption away from luminescence, because V${}_{\text{Ga}}$ is highly coupled to the lattice. This Franck-Condon shift is shown schematically in Figure 2d.

### 3.1. Total radiative output

**Figure 3:**
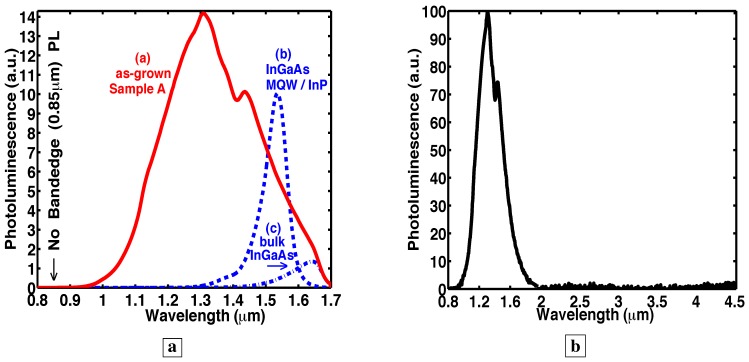
Room temperature PL [7]. **a,** PL from GaAs deep-centers and from InGaAs. Curve a shows PL from GaAs deep-centers in Sample A. We calibrated the internal radiative efficiency of these GaAs deep-centers to be about 90%. Curve b shows PL from high-quality InGaAs MQWs lattice-matched to InP. **b,** PL from GaAs deep-centers over a wide wavelength range.

The novel material shows a total radiative output which is at least comparable to high-quality InGaAs quantum-wells lattice-matched to InP. Figure 3a shows [7] room temperature PL from GaAs deep-centers and from InGaAs. The excitation in Figure 3a,b was a 10 mW HeNe laser. All data was normalized to a sample thickness of 0.25 *μ*m. Curve a in Figure 3a shows PL from the as-grown sample A of 2,488 Å of GaAs deep-centers. Below, we estimate a deep-center internal radiative efficiency of about 90%. Curve b in Figure 3a shows PL from sample D, the 21 periods of high-quality 120 Å InGaAs/120 Å InAlAs MQWs lattice-matched to indium phosphide (InP). Curve c in Figure 3a shows PL from sample E, the 2,325 Å of bulk InGaAs lattice-matched to InP. Significantly, sample A showed a total PL (integrated over wavelength) greater than from both high-quality InGaAs MQWs lattice-matched to InP (curve b in Figure 3a) and bulk InGaAs (curve c in Figure 3a).

In these photoluminescence (PL) studies, the epilayer thickness was always less than the characteristic absorption length of the excitation laser. Figure 5a indicates this by showing that the excitation creates minority holes throughout the entire deep-center layer.

### 3.2. Two measurements of radiative efficiency

The internal radiative efficiency was assessed in two ways [7]. First, the measured radiative-efficiency in the novel material was checked with a method reported by H. C. Casey [24,25,26,27]. Our measured PL were compared with our brightest samples of p-type GaAs (various thicknesses and concentrations of beryllium (Be) doped layers). Casey and Panish [25] and numerous others [24,26,27] have shown that the internal radiative efficiency of p-GaAs varies between 5% and 95%, and is a well-known function of the p-type doping. Thus, p-type GaAs has a radiative efficiency which is well calibrated and documented in the literature. We found that our brightest p-type GaAs calibration samples have internal radiative efficiencies which are in good agreement with the literature [24,25,26,27], and are thus ideal control samples. Second, we calibrated all elements of our optical setup. We directly measured the PL which is captured by a F/1.5 lens, and focussed onto a calibrated photodetector with a F/4 lens. We assumed that the externally measured PL consists [28,29] of only that portion of the internal PL radiation which is incident upon the sample surface at less than the critical angle. This gives a second estimate of the internal radiative energy. Both methods showed that sample A, the as-grown GaAs deep-centers (curve a in Figure 3a), had an internal radiative efficiency of slightly more than 90%. This internal efficiency describes radiation into *all* optical modes in *all* directions at *all* wavelengths and in *both* polarizations. (It is not the definition of internal efficiency in lasers, which describes radiation into a *single* optical mode at one *single* wavelength and *one* polarization.)

### 3.3. Evidence of a high-quality energy-gap having few nonradiative traps within the original bandgap

Figure 3b shows [7] room-temperature PL, obtained with a HeNe laser, from GaAs deep-centers (sample B) over a wider wavelength range. Significantly, no PL is observed at the bandedge (0.85 *μ*m) (Figure 3a,b, Figure 5b, Figure 7b) from any of the deep-center samples. For deep-centers in nominally n-type GaAs, this indicates an absence of free holes. The observed PL spectra are broad, and extend from 1.0 *μ*m to 1.9 *μ*m. This broad PL spectra results from transitions from states near the conduction-band (E${}_{\text{U}}$ in Figure 2b) to the many deep-acceptors (Si${}_{\text{Ga}}$-V${}_{\text{Ga}}$, V${}_{\text{Ga}}$, Si${}_{\text{As}}$, Si${}_{\text{Ga}}$-V${}_{\text{Ga}}$-Si${}_{\text{Ga}}$, and their ionization states) whose energies extend from the midgap down to the valence-band. Moreover, no PL is observed at wavelengths longer than 1.9 *μ*m. This absence of long-wavelength transitions is consistent with an absence of deep-acceptor states (which would act as either radiative and nonradiative traps) between the conduction-band and midgap. This absence of states is consistent with the observed large radiative efficiency. Our observations indicate that holes created by the excitation laser relax quickly from the valence-band to deep-states near midgap.

### 3.4. Absence of saturation of the photoluminescence

**Figure 4:**
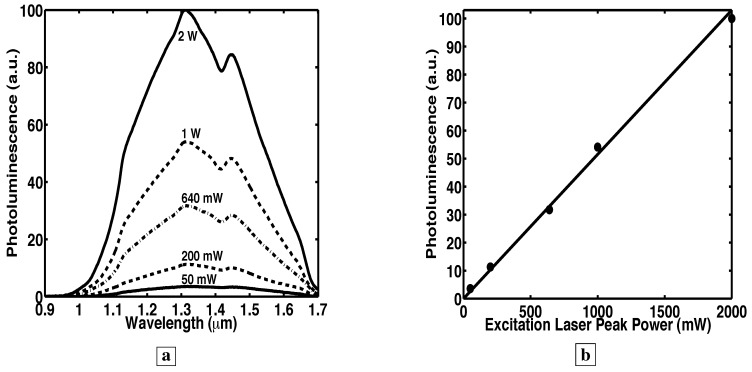
Room temperature PL [7] as a function of the optical pump power. **a,** The room temperature PL retains its spectral shape for all excitation laser peak powers up to 2 W. **b,** The PL peak at 1.31 *μ*m as a function of the excitation laser peak power.

Figure 4a shows room-temperature PL [7] from sample B, the 2,974 Å of GaAs deep-centers, as a function of the excitation peak power (from a few mW to 2 W). The excitation was a 816 nm GaAs laser having a 700 *μ*m × 200 *μ*m spot size. Significantly, the shape of the PL spectra in Figure 4a remains unchanged for all excitation intensities. Two peaks (at 1.31 *μ*m and 1.45 *μ*m) are always observed, and the relative heights of the two peaks remain unchanged for all excitation intensities. Figure 4b shows that the PL peak at 1.31 *μ*m increases linearly with excitation intensity, even up to 2 W. Thus, even with a 2 W optical excitation (equivalent to 1.1 kW/cm${}^{2}$), we were unable to saturate the deep-level transitions. Thus, with increasing optical excitation, the PL spectra retain the same shape, and the PL increases linearly.

**Figure 5:**
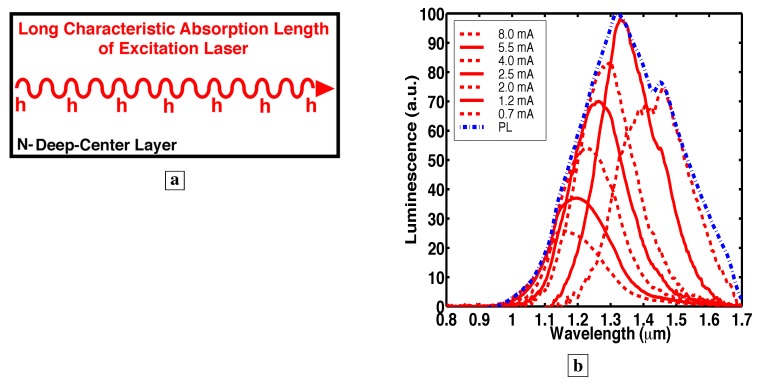
Characteristic absorption length of the optical pump and inhomogeneous broadening of the PL [7]. **a,** The excitation laser creates minority holes throughout the deep-center layer because the characteristic absorption length of the laser is greater than the deep-center layer thickness. **b,** PL spectrum (dashed-dotted line), on top of which is superimposed several normalized EL spectra (solid and dashed lines).

### 3.5. Spectral shift of the electroluminescence (EL) and inhomogeneous broadening of the PL

In sharp contrast to the PL, the EL spectral shape changes significantly with injection, as shown [7] in Figure 5b (and Figure 8a). Figure 5b shows the PL spectrum (dashed-dotted line), on top of which is superimposed several EL spectra (solid and dashed lines), for sample C. The EL spectra have been normalized so that the EL peaks lie on top of the PL spectrum. Figure 5b shows that the EL at any specific current excites only a subset of the transitions (wavelengths) in the original PL spectrum. Moreover, as the current is incrementally increased, the EL spectrum shifts incrementally to shorter wavelengths. The latter indicates that the exact value of the current can be used to select specific transitions (wavelengths). This indicates inhomogeneous broadening of the PL.

**Figure 6:**
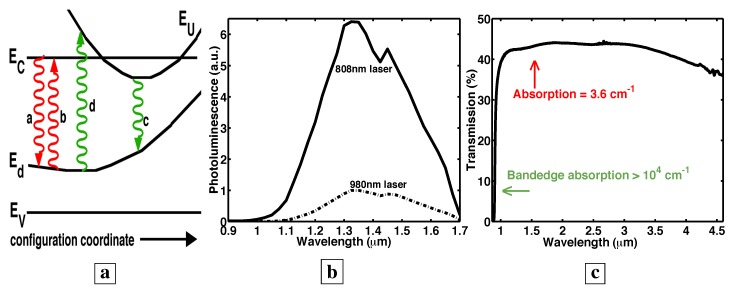
The donor-V${}_{\text{Ga}}$ complex is known [8] to show a Franck-Condon shift [7]. **a,** Arrows c and d show a Franck-Condon spectral shift of absorption away from luminescence. **b,** Photoluminescence [7] at different excitation wavelengths from the GaAs deep-centers. The excitation at 808 nm (solid curve) yields much brighter PL than the excitation at 980 nm (dashed curve). The new material absorbs efficiently only at short wavelengths (<980 nm), whereas the PL occurs at long wavelengths (1-1.7 *μ*m). **c,** The measured transmission [7] indicates that, at wavelengths (1–1.7 *μ*m) of bright PL from the GaAs deep-centers, the absorption loss is very small.

### 3.6. A Franck-Condon shift

It is well-known [7,11,19,20,21,22,23] that n-type GaAs is compensated by donor-vacancy-on-gallium (donor-V${}_{\text{Ga}}$) complexes under As-rich conditions. It is also well known [7,8,11,13,17,18] that the donor-V${}_{\text{Ga}}$ complex shows a Franck-Condon spectral shift of absorption away from luminescence, because V${}_{\text{Ga}}$ is highly coupled to the lattice. (Arrows a and b in Figure 6a show luminescence and absorption at the same energies. Arrows c and d in Figure 6a show a Franck-Condon shift where absorption occurs at higher energies than luminescence. The literature [8] says that the upper state E${}_{\text{U}}$ corresponds to a state centered on the donor in the donor-V${}_{\text{Ga}}$ complex, whereas the lower state E${}_{\text{d}}$ corresponds to a state centered on the V${}_{\text{Ga}}$ in the complex. Vacancies are highly coupled to lattice-vibrations. The configuration coordinate in Figure 6a describes the coupling of vacancies to lattice-vibrations.) We now show that both transmission and photoluminescence at different excitation wavelengths are consistent with this well-known Franck-Condon shift.

### 3.7. Photoluminescence at different excitation wavelengths shows that absorption occurs at shorter than 1 μm, but emission occurs at longer than 1 μm

Figure 6b shows the PL [7] from the GaAs deep-centers at two different excitation wavelengths. The excitation at 808 nm (solid curve) yields much brighter PL than the excitation at 980 nm (dashed curve). All data in Figure 6b correspond to the same number of incident photons. The PL from different excitation wavelengths (i.e., photoluminescence excitation) is often used as a measure of absorption. Thus, Figure 6b shows that efficient absorption in the novel material occurs only at short wavelengths (<980 nm), whereas the PL occurs at long wavelengths (1-1.7 *μ*m). This is consistent with the well-known Franck-Condon shift associated with V${}_{\text{Ga}}$-complexes.

### 3.8. Transparency in the novel material is achieved at near-zero injection

Figure 6c shows that [7], at wavelengths (1-1.7 *μ*m) of bright PL from the GaAs deep-centers, the material is nearly transparent even at zero injection. The measured transmission through the GaAs deep-centers in Figure 6c indicates an absorption loss of less than 3.6 cm${}^{-1}$ at 1.6 *μ*m wavelengths. This absorption loss of 3.6 cm${}^{-1}$ is considerably less than the typical bandedge absorption (10${}^{4}$ cm${}^{-1}$). Thus, the injection which achieves transparency in the novel material is much less than in direct-gap semiconductors. Figure 6c also shows that, in the novel material, absorption occurs at short wavelengths (<1 *μ*m), whereas the PL occurs at long wavelengths (1-1.7 *μ*m). Again, this is consistent with the well-known Franck-Condon shift associated with V${}_{\text{Ga}}$-complexes.

### 3.9. The new material has PL showing a high degree of temperature insensitivity

**Figure 7:**
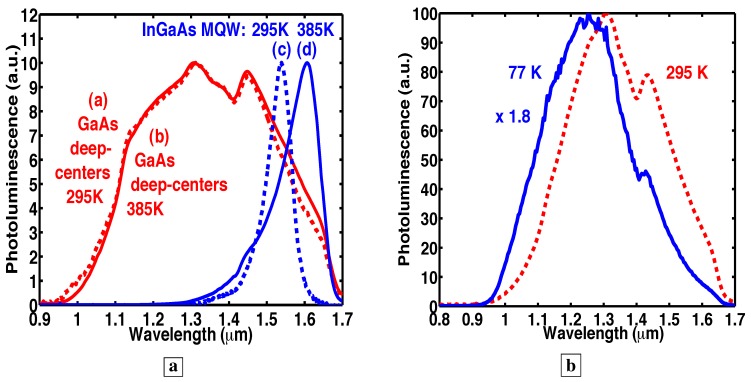
**a,** The PL [7] from GaAs deep-centers and high-quality InGaAs MQWs at 295 K (dashed lines) and 385 K (solid lines). **b,** The PL of GaAs deep-centers at 295 K (dashed line) and 77 K (solid line). All PL have been normalized to the peak values.

It is well known that the optical-emission from high-quality MQWs changes dramatically with temperature. The latter results from the strong temperature dependence of the bandgap energy in conventional semiconductors. In stark contrast to conventional semiconductors, we report virtually no change in both the spectral shape and peak height of the PL [7] from the GaAs deep-centers between 295 K (curve a [dashed curve] in Figure 7a) and 385 K (curve b [solid curve] in Figure 7a). For comparison, the PL peak from high-quality InGaAs MQWs shifts from 1.53 *μ*m to 1.60 *μ*m between 295 K (curve c [dashed curve] in Figure 7a) and 385 K (curve d [solid curve] in Figure 7a). This is accompanied by a drop (not shown) in the InGaAs PL peak at 385 K to 0.7 times the PL peak at 295 K. Figure 7b shows that the PL (normalized to the peak) from the GaAs deep centers at 77 K (solid curve) shifts to slightly longer wavelengths at 295 K (dashed curve). The PL at 77 K was 1.8 times that at room temperature.

### 3.10. Electroluminescence spectra from p-n junction

**Figure 8:**
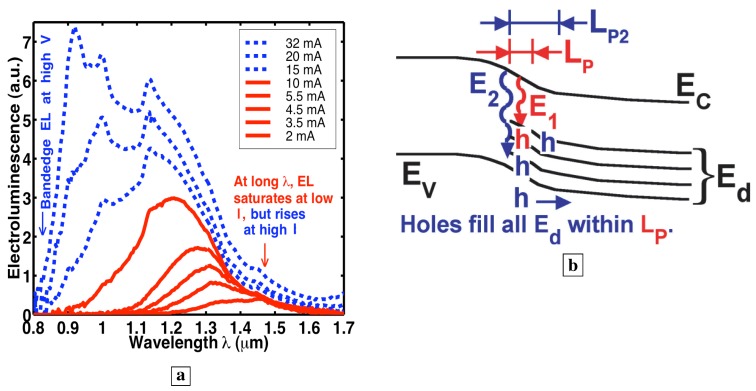
**a,** Room temperature EL spectra [7] of p-n junction with the deep-center-layer as the n-region. **b,** Energy band diagram showing the p-layer on the left and the n-type deep-center layer on the right. At low injection, free holes are captured onto deep-centers within one L${}_{P}$ of the p-n junction, and scatter up to midgap states. (Holes are labeled in red). At higher injection, the additional holes fill all deep-states within one L${}_{P}$ of the p-n junction from the midgap down to the valence-band. As these deep-states fill up, the EL saturates at the long wavelengths corresponding to transitions to these deep-states.

Figure 8a shows room temperature EL spectra [7] from a p-n junction where the n-layer is the deep-center-layer. Details of the EL allow us to evaluate some important lifetimes in the material. We show below that the blue-shift of the EL in Figure 8a relative to the PL (Figure 3) results from the small volume (one L${}_{P}$ into the deep-center-layer) over which free holes exist in Figure 8a. This is equivalent to a fast lifetime for capture of free holes by deep-centers.

### 3.11. Electroluminescence in the absence of a p-layer

**Figure 9:**
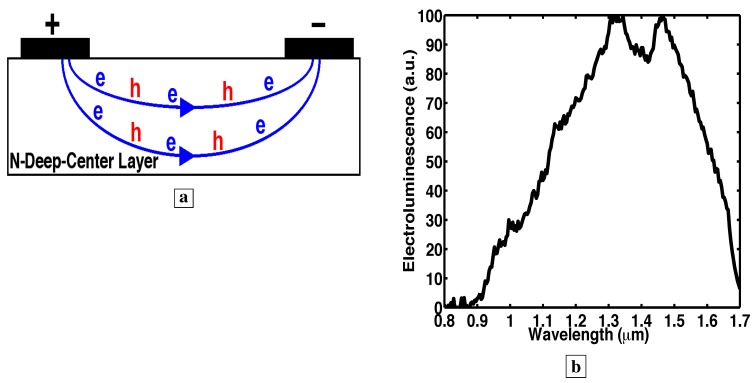
Room temperature EL [7] from a device which does not have a p-layer. **a,** In a device consisting of only the n-type deep-center-layer, holes are created via impact ionization of electrons over a large volume of the deep-center-layer (all along the electron paths). **b,** The EL spectra from the deep-center-layer looks a lot like the PL when the holes are created in a large volume of the deep-center-layer (all along the electron paths). This is unlike Figure 8a, where the EL spectra exhibits a spectral blue-shift relative to the PL, and where the holes in Figure 8a exist only in a small volume (the first L${}_{P}$) of the deep-center-layer.

A useful control sample [7] is shown in Figure 9. This device consists of only the n-type deep-center-layer with no p-layer. Measurements with this device were useful because the mechanism for hole injection into the deep-center layer differs significantly from that in a p-n junction. Without a p-layer, holes are created in the deep-center-layer via impact ionization of majority electrons all along the electron paths. The latter occupy most of the volume of the entire deep-center layer. When the holes are created over a large volume (Figure 9a), the EL spectral shape (Figure 9b) looks a lot like the PL (Figure 3). This makes sense because, in PL, holes are also created over a large volume (the entire deep-center-layer). The active volume in PL is indicated in Figure 5a by an epilayer thickness which was always less than the characteristic absorption length of the excitation laser.

### 3.12. Possible explanations for blue-shift of EL spectra from p-n junction: heating or internal electric fields?

Figure 9 and Figure 7a show [7] that the blue-shift of the EL in Figure 8a cannot be explained by either device heating or a Stark effect due to an internal electric field. The voltage and current used for electron-injection to obtain the EL in Figure 9b are somewhat larger than those used in the p-n junction EL of Figure 8a. This is significant because any I-V heating would be greater in Figure 9 than in Figure 8a. Moreover, the electric field across the deep-center-layer (and any Stark effect) is greater in Figure 9 (15 V drop) than in Figure 8a (5.5 V drop). Since the EL in Figure 9b and Figure 8a incur similar I-V heating and internal electric field (and Stark effect), then heating and electric field (and Stark effect) cannot explain the spectral blue shift in the p-n junction EL of Figure 8a relative to the EL of Figure 9b and to the PL of Figure 3. This is consistent with our earlier observation in Figure 7a that the PL at 385 K is virtually the same as the PL at 295 K.

### 3.13. Absence of bandedge emission and absence of free holes

Significantly, no bandedge PL (0.85 *μ*m) is observed [7] in Figure 3a,b, Figure 5b, Figure 7b. The absence of bandedge PL from the n-type deep-center-layer indicates an absence of free holes. The latter indicates that free holes are quickly trapped by deep-centers before a conduction-to-valence-band transition occurs. The lifetime $\tau_{dv,h}$ for hole capture into a deep-center is indicated in Figure 1b. The hole-diffusion-length L${}_{P}$ is related to $\tau_{dv,h}$ through, $L_{P}^{2}=\left(k_{B}T/q\right)\;\mu_{h}\;\tau_{dv,h},$ where $\mu_{h}$ is the hole mobility in the deep-center-layer. A fast $\tau_{dv,h}$ implies a short L${}_{P}$. This has important consequences for EL. Figure 8b shows that the electrically injected holes from the p-region are immediately captured by deep-centers in the first L${}_{P}$ of the n-type deep-center-layer.

### 3.14. Evidence of a small hole diffusion length in the deep-center layer

The EL spectra in Figure 8a can be explained [7] by a small fixed value of L${}_{P}$, and the small number of deep-centers within a small L${}_{P}$. At low injection, holes scatter up to deep-levels near midgap within one L${}_{P}$ of the junction. At higher injection, holes have filled all states near midgap within one L${}_{P}$ of the junction, and holes start to populate deep-levels closer to the valence-band within one L${}_{P}$ of the junction. This makes possible transitions involving higher photon energy (E${}_{2}$ as well as E${}_{1}$ in Figure 8b), as electrons combine with holes located at deep-levels closer to the valence-band. Thus, at higher injection, the EL spectra in Figure 8a shift to shorter wavelengths.

The solid curves in Figure 8a show that the EL at longer than 1.32 *μ*m increases for small injection, but saturates at a low current. For example, the EL at 1.45 *μ*m in Figure 8a remains the same for all injection between 2 mA to 10 mA. This saturation of the EL brightness at long wavelengths can be explained by a filling with holes of all midgap states within one L${}_{P}$ of the p-n junction. Since additional holes at higher injection no longer reach midgap states, the number of transitions to midgap states remains the same, and the EL at long wavelengths saturates at these injection levels.

At currents greater than 10 mA (dashed in Figure 8a), the EL at longer than 1.32 *μ*m, surprisingly, starts to rise again. This increase in the long-wavelength EL is accompanied by the presence of free holes: bandedge EL (0.85 *μ*m) is observed only for injections greater than 10 mA in Figure 8a. This is sensible because, when most deep-centers within one L${}_{P}$ of the junction have captured a hole, the repulsive Coulomb force makes it difficult for additional holes to be captured by the same deep-centers. Thus, at these higher currents, some free holes exist within one L${}_{P}$ of the junction (Figure 8b), and these free holes can give rise to bandedge EL. The latter holes can also be trapped into unoccupied states near midgap further from the junction (L${}_{P2}$>L${}_{P}$ in Figure 8b) in the interior of the deep-center layer. This explains why bandedge EL (dashed in Figure 8a) occurs simultaneously with a sudden rise in the EL at long wavelengths (1.32-1.7 *μ*m) beyond the saturated values at low current.

**Figure 10:**
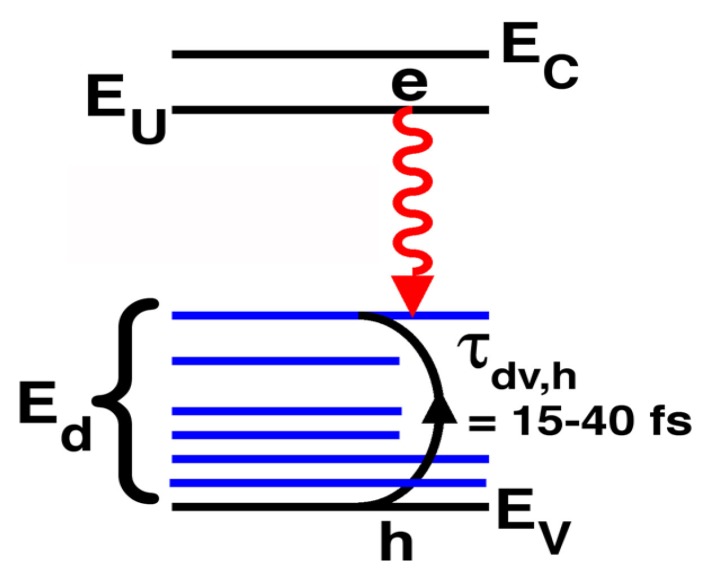
This is an ideal optical material [7]: the four-level system has a large radiative output, and exhibits fast depopulation of electrons from the lower state of the optical transition.

### 3.15. Fast depopulation of the lower-state of the optical transition

Previously [7,30,31], using estimates of the hole diffusion length in the deep-center material, we showed that the hole capture into (i.e., the depopulation of electrons out of) the lower state of the optical-transition is very fast (10–100 fs) at room-temperature. This hole capture lifetime is consistent with previous measurements [10] of the coefficient for capture of a free hole onto the native deep-acceptors (mainly V${}_{\text{Ga}}$ and their complexes) in n-type GaAs. This lifetime for depopulation of the lower state is also consistent with previous direct measurements [32,33] (via fast pump-probe experiments) of the 100 fs trapping of holes by the vacancy-on-Ga-site. The physics which explains the fast hole capture onto deep-centers is that, in compensated semiconductors, the deep-acceptor complexes are negatively charged, and thus exhibit a large capture cross-section for positively-charged holes. Thus, we demonstrated that the novel material constitutes a four-level system which shows both a bright optical-transition and fast depopulation of the lower state of the optical-transition. This is summarized in Figure 10. Such a four-level system is known [34,35] to be an ideal optical material for lasers.

### 3.16. Summary

Thus far, we have shown that the deep-centers in the novel material showed a total radiative output which is at least comparable to that from the same thickness of high-quality InGaAs-quantum-wells-on-InP, and a large internal-radiative-efficiency for emission into all wavelengths longer than the bandgap. This indicates that a new high-quality energy gap has formed between the conduction-band and about midgap. Radiative emission is observed at energies greater than half-the-bandgap. The well-known Franck-Condon spectral shift was observed via both transmission measurements and photoluminescence at different excitation wavelengths. Moreover, the deep-centers showed very temperature-insensitive photoluminescence over a rise of 90 ${}^{\circ }$C above room-temperature. We also found that the PL is inhomogeneously broadened. An important aspect of our work was the observation of a significant difference between photoluminescence and electroluminescence. In our work, the PL could not be saturated, and the PL spectra retain the same shape for all optical pump powers. In stark contrast, the EL is found to saturate at long wavelengths, and to show a strong spectral blue-shift with increasing injection. These observations were explained by considering the number of deep-centers which are probed in PL versus EL. Since the characteristic absorption length of the pump laser was greater than the deep-center layer thickness, the PL probes a large volume, the entire deep-center layer, and thus, a large number of deep-centers. With a p-n junction, the EL probes only a small volume, the first L${}_{P}$ of the deep-center layer, and thus, only a small number of deep-centers. Our observation of a small L${}_{P}$ indicates fast capture of free holes onto deep-centers. This fast capture of free holes onto deep-centers is consistent with the absence from all our deep-center samples of bandedge emission in PL. Finally, the novel material is found to be ideal for lasers, with fast femtosecond depopulation of the lower state of the optical transition.

## 4. Stimulated Emission and Laser Action

### 4.1. Regimes of behavior in the L-I curve

Solution of the laser rate equations [34,36] shows three regimes of behavior. At low injection, spontaneous-emission (also known as fluorescence or light-emitting-diode (LED) behavior) is indicated [34,36] as a +1 slope in a log-log plot of optical-emission as a function of current density J (the “L-I” curve). This indicates that the optical-emission is proportional to the first power of J. At a higher injection, stimulated-emission is observed, and rises much more quickly as J${}^{s}$, where s>1. On a log-log plot, stimulated-emission shows [3,4,34,36,37,38,39] a superlinear slope *s*, (s>1). Typical values of the superlinear slope *s* range from 2.5-3.5, for large microdisk lasers [37,38] at room-temperature, to 2.9-11, for very small microdisk lasers [3,4,39] at low temperature. This superlinear growth of the optical-emission continues until the photon number reaches 1/*β* [36], where *β* is the spontaneous emission coefficient, beyond which the laser output increases linearly with J. This linear dependence of the L-I curve at high injection indicates a pinning of the population inversion at its threshold value. At threshold, the slope of the L-I curve on a log-log plot has its greatest value [34].

## 5. Stimulated Emission

### 5.1. Superlinear L-I at specific wavelengths

One criterion for demonstrating stimulated-emission is identified in three classic papers which reported the first demonstration of stimulated-emission in GaAs [40], bulk GaN [41], and GaN microdisks [38]. The key is that, in situations where laser action is not achieved, the stimulated-emission shows both a spectral shift and broadening with increasing injection. No Fabry-Perot modes are observed. (See Figure 3 in [40], Figure 2 in [41], and Figures 1 and 2 in [38].) Thus, in the absence of laser action, the criteria for stimulated-emission was not the observed spectral broadening nor the absence of Fabry-Perot modes. Rather, the criterion for stimulated-emission was a superlinear L-I curve at a *specific* wavelength: see Figures 1 and 2 in [38]. This is especially true in dye lasers, where the stimulated-emission spectrum is broad: see Figure 8 in [42]. The superlinear L-I curve must be demonstrated at a *specific* wavelength because, in stimulated-emission, a photon gives rise to additional photons at the *same* wavelength.

**Figure 11:**
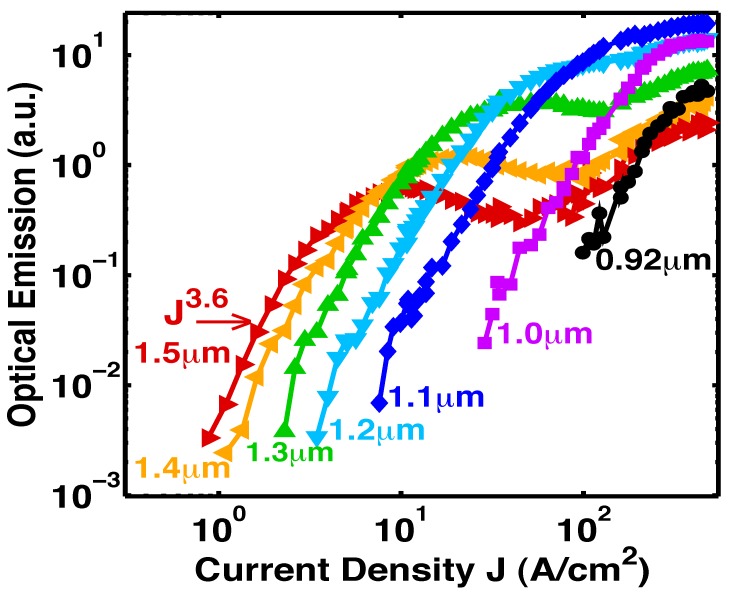
Room-temperature stimulated-emission measured from the edge of Sample F [6]. The superlinear rise of the optical-emission as J${}^{3.6}$ is the signature of stimulated-emission.

Figure 11 shows the optical-emission [6] measured from the sample edge (edge emission) as a function of J at room-temperature at specific wavelengths. At every wavelength, and for a significant range of J, the optical-emission is seen to increase by two to three orders-of-magnitude with a fast rise of J${}^{3.6}$. This superlinear rise of the optical-emission as J${}^{s}$ at specific wavelengths is the signature of stimulated-emission. Note that, as the injection is increased, the stimulated-emission can be tuned widely from long wavelengths (about half-the-bandgap) to short wavelengths (near the bandgap). (This observed wavelength tuning range (900–1,600 nm) was limited by the response of our photodetector. The actual tuning range may be a bit wider.) In Figure 11, also note that, as the stimulated-emission at shorter wavelengths rises, the stimulated-emission at longer wavelengths clamps.

**Figure 12:**
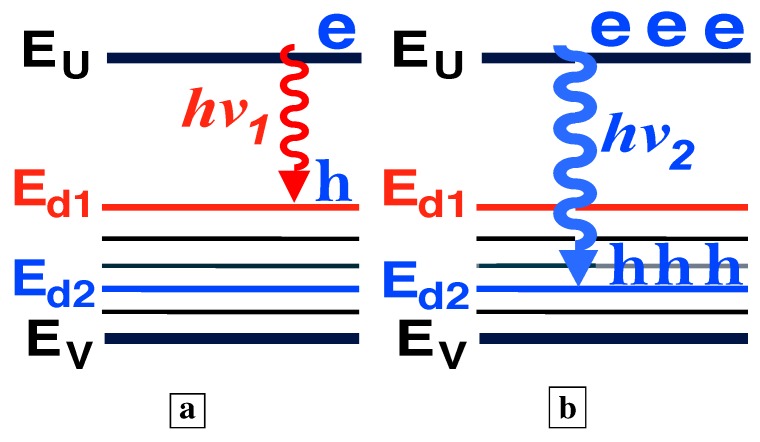
Electron and hole population distributions which explain both the shift to shorter wavelengths of the stimulated-emission (the superlinear L-I) and the clamping of the long-wavelength emission in Figure 11 with increasing injection. **a,** At small injection, holes scatter up to midgap states. **b,** At higher injection, the large number of holes at energies (e.g., E${}_{\text{d2}}$) further down from the midgap dramatically increases the shorter-wavelength (e.g., $h\nu_{2}$) optical-emission rate. Holes recombine radiatively (e.g., $h\nu_{2}$) with electrons before they can scatter up to midgap states. The optical-emission at $h\nu_{2}$ “uses up” the holes needed for long-wavelength emission.

### 5.2. Carrier population distribution and the L-I curve

Figure 12 explains both the shift to shorter wavelengths of the stimulated-emission (the superlinear L-I) and the clamping of the long-wavelength emission in Figure 11 with increasing injection. Figure 12a shows that, at small injection, holes scatter up to midgap states, and stimulated-emission occurs at long wavelengths. Figure 12b shows that, at higher injection, many holes arrive at energies (e.g., E${}_{\text{d2}}$) further down from the midgap and closer to the valence band. The large number of holes at these energies (e.g., E${}_{\text{d2}}$) dramatically increases the shorter-wavelength (e.g., $h\nu_{2}$) optical-emission rate. Hence, holes recombine radiatively (e.g., $h\nu_{2}$) with electrons before they can scatter up to midgap states. Thus, the shorter-wavelength (e.g., $h\nu_{2}$) optical-emission processes “use up” the holes needed for longer-wavelength (e.g., $h\nu_{1}$) optical emission. This explains the shift to shorter wavelengths of the stimulated-emission in Figure 11, as the long-wavelength optical-emission clamps.

### 5.3. The Bernard-Duraffourg criterion

**Figure 13:**
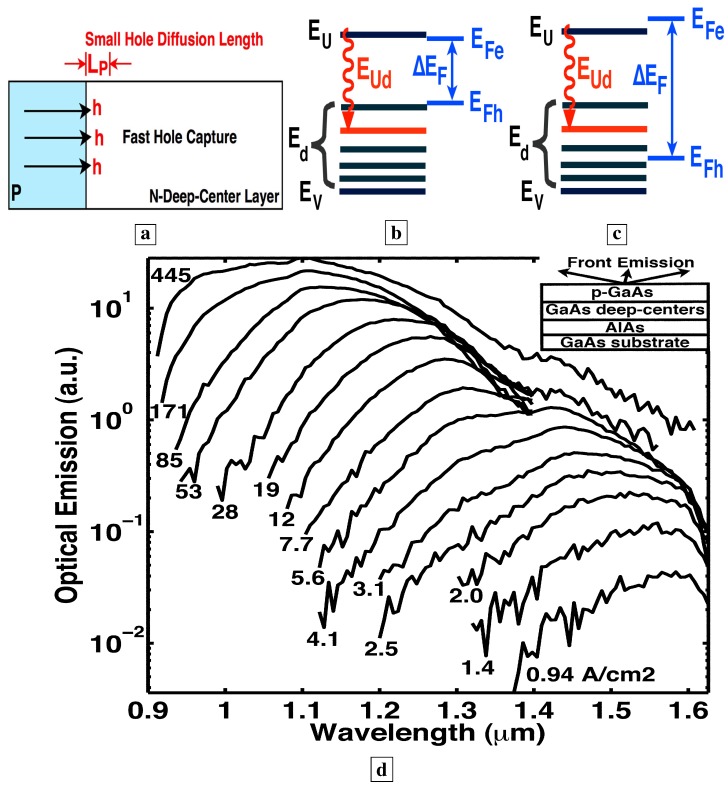
Room-temperature spectra measured from the surface of Sample F [6]. **a,** Layer structure showing p-doped layer, n-type deep-center layer, and hole diffusion length. **b,** At low current, the hole quasi-Fermi level $E_{Fh}$ is above midgap, and most of the optical emission is at long wavelengths (1.6 *μ*m). At high current, $E_{Fh}$ is pulled far below midgap, and most of the optical emission is at shorter wavelengths (1.1 *μ*m). Here, $\Delta E_{F}$ exceeds the transition energy $E_{Ud}$. This is the Bernard-Duraffourg signature for stimulated emission at $E_{Ud}$. **d,** Room-temperature optical-emission spectra measured from the sample surface at different injection in the absence of a resonant cavity. The observed blue shift in the optical emission corresponds to a similar increase in $\Delta E_{F}$ with increasing injection.

Another criterion for demonstrating stimulated emission between two energy bands is a sufficiently large quasi-Fermi level separation, as derived in a classic paper by Bernard and Duraffourg [43]. Our previous work [6,7] showed that, as the injection increases, the hole quasi-Fermi-level within the first L${}_{P}$ of the deep-center layer drops from near midgap to near E${}_{\text{V}}$, as indicated in Figure 13a–c. This is manifest in the measured spectra, Figure 13d, as a shift of the optical-emission from half the bandgap energy (1.6 *μ*m) to shorter wavelengths (1.0 *μ*m) near the bandgap. The observed spectral blue shift of the optical emission corresponds to a similar increase in the separation between the electron and hole quasi-Fermi levels ($E_{Fe}$, $E_{Fh}$) with increasing injection. The increase of the quasi-Fermi level separation $\Delta E_{F}$ with increasing injection is a signature of stimulated emission, as demonstrated in a classic paper [43]. The latter showed that when $\Delta E_{F}$ exceeds the transition energy $E_{Ud}$, the stimulated emission exceeds the absorption at $E_{Ud}$. Figure 13d shows that $\Delta E_{F}$ increases with increasing injection. Consistent with Figure 13d [6] and the Bernard-Duraffourg result, Figure 11 shows that the superlinear L-I (stimulated emission) is achieved at shorter wavelengths, as $\Delta E_{F}$ exceeds the transition energies associated with shorter wavelengths, with increasing injection. This is the Bernard-Duraffourg signature of stimulated emission, and is depicted in Figure 13b-c.

### 5.4. Increasingly superlinear L-I with a resonant cavity

Figure 11 shows that the onset of stimulated-emission occurs at a J less than 1 A/cm${}^{2}$ (at wavelengths longer than 1.3 *μ*m). This stimulated emission was observed in the *absence* of a resonant cavity or waveguide. Any enhancement due to cavity effects was avoided by deliberately etching mesa edges with a random roughness. With a longer optical path and higher quality optical confinement, we would expect the stimulated-emission to increase, and the exponent *s* in the functional dependence J${}^{s}$ of the stimulated emission to be larger. Indeed, Figure 15 shows [5] this to be true: the superlinear exponent s is about 3 *without* a resonant cavity (Figure 11), and is 64 *with* a resonant cavity (Figure 15a). The resonant cavity is formed by locating a distributed-Bragg-reflector (DBR) under the active layer, and reactive ion etching (RIE) a pixel to a depth of 4.5 DBR periods, as shown in Figure 1e.

### 5.5. Observation of a gain larger than a significant loss

A final piece of evidence for stimulated-emission is the observation [5,6] of an optical gain large enough to overcome a significant loss. In the next section, when we discuss laser action, we will also discuss both the nature of the optical modes and the optical emission spectra from deep-centers in the presence of a resonant cavity. Here, we will summarize the results, and defer the more detailed explanations until the next section. In the presence of a resonant cavity, we would expect that the largest spectral peak in the optical emission to correspond to a low-loss optical mode. It is thus both surprising and significant that the spectra [5] in Figure 17c, Figure 18b, and Figure 19c below all show the low-loss total-internal-reflection (TIR) mode, labeled G, to be suppressed, while the very lossy vertical mode, labeled F, dominates the spectra as a narrow peak. The latter signifies that enough material gain exists to overcome the large optical loss, which we will find to be a 70% transmission loss with each reflection of the lossy vertical mode at the sample surface. This large optical gain, which is needed to overcome the large loss, is another indicator of stimulated-emission.

### 5.6. Summary

Thus far, we have demonstrated stimulated-emission from deep-centers in highly n-doped GaAs, and electrical injection was the pump mechanism. The evidence for stimulated-emission includes: a superlinear L-I curve, a quasi-Fermi level separation large enough to satisfy the Bernard-Duraffourg criterion, and an optical gain large enough to overcome significant loss. We have demonstrated that the room-temperature stimulated-emission from GaAs deep-centers can be tuned very widely from the bandgap to half-the-bandgap by changing the electrical injection. Room-temperature deep-center stimulated-emission is demonstrated at electrical injections less than 1 A/cm${}^{2}$. This small injection which achieves stimulated-emission will be explained below by the fast capture of free holes onto deep-centers.

**Figure 14:**
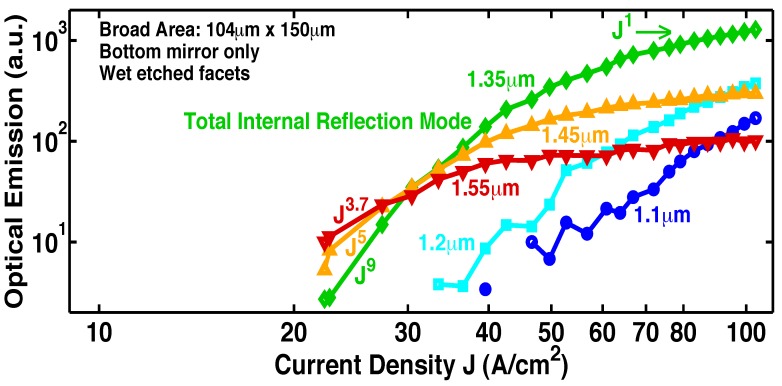
Room-temperature L-I curves [5] measured in cw-mode from the same pixel on Sample G at different wavelengths in a single-pass geometry. At current densities greater than 65 A/cm${}^{2}$, the optical-emission at 1.35 *μ*m becomes proportional to the first power of J, and the longer wavelength (e.g, 1.45 *μ*m and 1.55 *μ*m) optical-emission clamps at a constant value. The latter indicates that, at these injection levels, all the additional carriers supply the optical emission into the low-loss total-internal-reflection mode, rather than the longer wavelength lossy modes. These two observations indicate gain pinning and single-pass laser action.

## 6. Laser Action

### 6.1. Evidence for laser action

Our evidence [5] for laser action from GaAs deep-centers is summarized here: (1) L-I curves, showing a superlinear regime at low injection, where the stimulated-emission rises three orders-of-magnitude with the functional dependence J${}^{s}$, and a regime linear in J at higher injection, indicating gain-pinning and laser action. The exponent *s* in the stimulated-emission regime is found to be larger for longer optical paths and higher quality optical confinement. (2) In the single pass geometry of Sample G below, laser action is observed for the longest wavelength total-internal-reflection (TIR) mode of Figure 1g. At an injection high enough for laser action in this TIR mode, the optical emission from lossy modes, such as the vertical mode of Figure 1f, is clamped at a constant value. See Figure 14. (3) With a resonant cavity and RIE facets, we observed a pinning of the carrier distribution among the energy-levels at all injections greater than threshold. Without a resonant cavity, previous work [6,7] showed that an increasing injection results in a marked shift in the carrier distribution, and a rise in shorter wavelength emission. (4) With a resonant cavity and RIE facets, the dominant mode in the optical emission spectrum (Figure 17c, Figure 18b, and Figure 19c) is the lossy vertical mode of Figure 1f. In order to be dominant, this lossy mode must undergo significant optical gain. A significant gain is another indicator of laser action.

### 6.2. Relevant optical modes

Figure 1d shows a single-pass measurement from the edge of Sample G. A bottom DBR was added in Sample G to increase the optical path for resonant normal wave vectors K${}_{\text{Z}}$. Wet-etched rough facets preclude the optical-feedback characteristic of resonant cavities. Figure 1f shows the “vertical” waveguide mode, which is a mode that reaches the sample surface at normal incidence and whose longitudinal wave vector K${}_{\text{X}}$ is nearly zero. This vertical mode is very lossy, because 70% of the power reaching the sample surface is transmitted vertically, and lost from the waveguide. Figure 1g shows the longest wavelength total-internal-reflection (TIR) mode. Here, rays from within the semiconductor are incident upon the sample surface at the critical angle $\theta_{\text{C}}$ for TIR. Figure 1h shows shorter wavelength TIR modes. Here, rays from within the semiconductor are incident upon the sample surface at an angle greater than $\theta_{\text{C}}$. These shorter wavelength modes make fewer passes through the active region. The vertical mode of Figure 1f makes the largest number of passes through the active region, but is quite lossy.

### 6.3. Increasingly superlinear L-I with better optical confinement

Sample F was *not* placed within a resonant cavity or waveguide. Figure 11 [6] shows that the single-pass L-I curves from Sample F rise a significant two to three orders-of-magnitude at every wavelength at a superlinear rate of J${}^{3}$. This superlinear rise as J${}^{3}$ is the signature of stimulated-emission. We found that the superlinear slope *s* of the stimulated-emission is larger for longer optical-paths.

### 6.4. Single-pass geometry having a long optical path

**Figure 15:**
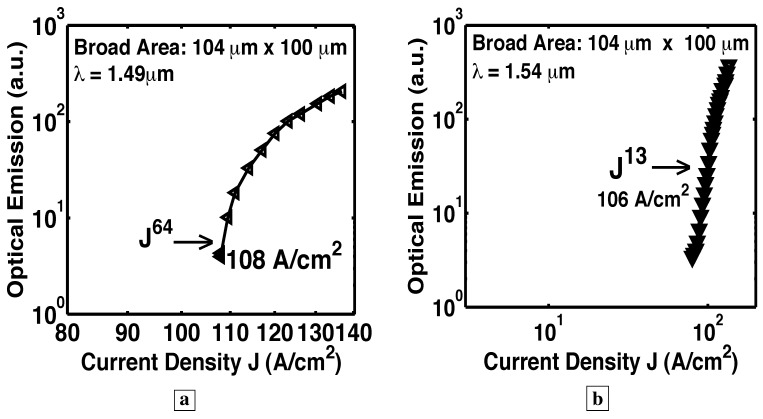
Room-temperature L-I curve [5] measured in cw-mode at a fixed wavelength from a resonant cavity with both a bottom DBR and RIE facets. The pixel has been labeled by its threshold current density (where the log-log plot of L-I shows the greatest slope), and the functional form J${}^{s}$ of the stimulated emission.**a,** With the optical-feedback resulting from RIE facets, the stimulated-emission from Sample H is seen to rise even more sharply as J${}^{s}$, where *s* is as large as 64. **b,** Sample J shows a “S-shaped" L-I curve, which indicates a transition from spontaneous-emission, to stimulated-emission, towards laser-action.

**Figure 16:**
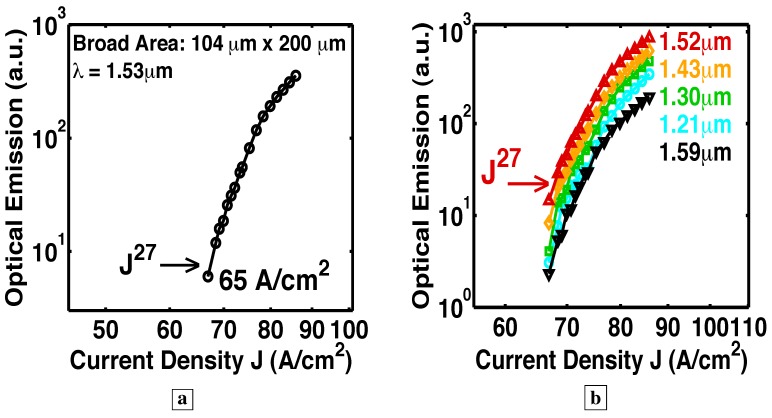
Room-temperature L-I curves [5] measured in cw-mode from a resonant cavity with both a bottom DBR and RIE facets. **a,** With the optical-feedback resulting from RIE facets, the stimulated-emission from Sample K is seen to rise sharply as J${}^{s}$, where *s* is as large as 27.**b,** The constant vertical separation on a log-log plot of the L-I curves at different wavelengths from the same pixel indicates that the optical-emission has the same spectral shape for all indicated J, and that the carrier distribution is pinned among the energy-levels. (Without the resonant cavity, the optical emission shows a significant shift in the population distribution with increasing injection.)

**Figure 17:**
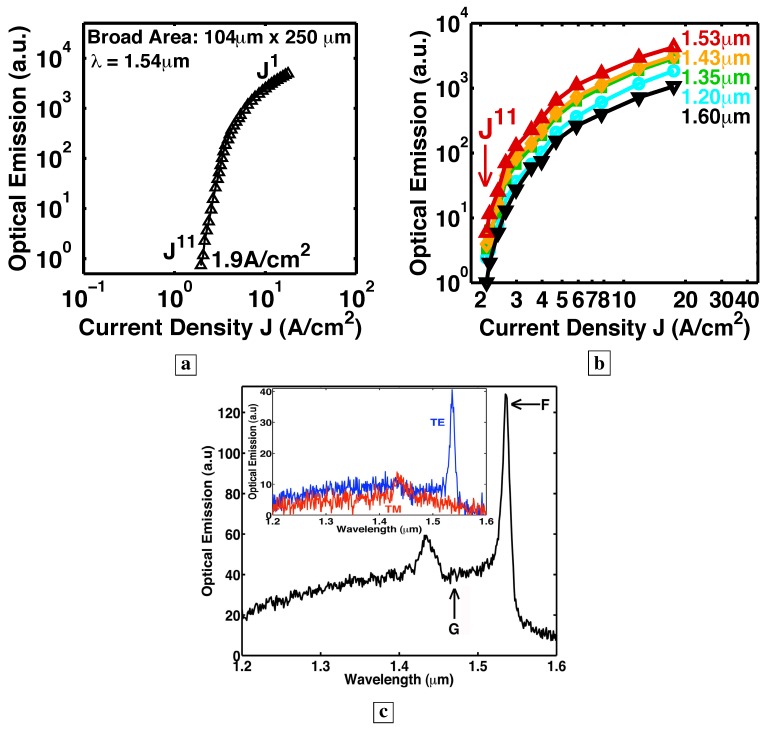
Room-temperature measurements [5] in cw-mode from a resonant cavity (bottom DBR and RIE facets). **a,** The stimulated-emission at 1.54 *μ*m from Sample L is seen to rise a significant three orders of magnitude as J${}^{11}$, beyond which the optical-emission quickly becomes linear in J. The latter indicates gain pinning and laser action. **b,** The constant vertical separation on a log-log plot of the L-I curves at different wavelengths from the same pixel indicates that the optical-emission has the same spectral shape for all indicated J, and that the carrier distribution is pinned among the energy-levels. (Without the resonant cavity, the optical emission shows a significant shift in the population distribution with increasing injection.) **c,** Optical-emission spectra from Sample L at 17 A/cm${}^{2}$. All samples having a resonant cavity showed that the low-loss TIR mode, labeled G in Figure 17c, is suppressed, while the lossy vertical mode, labeled F in Figure 17c, dominates the spectra as a narrow peak. The latter signifies that enough material gain exists to overcome the large 70% transmission loss incurred by the vertical mode with each trip to the sample surface.

**Figure 18:**
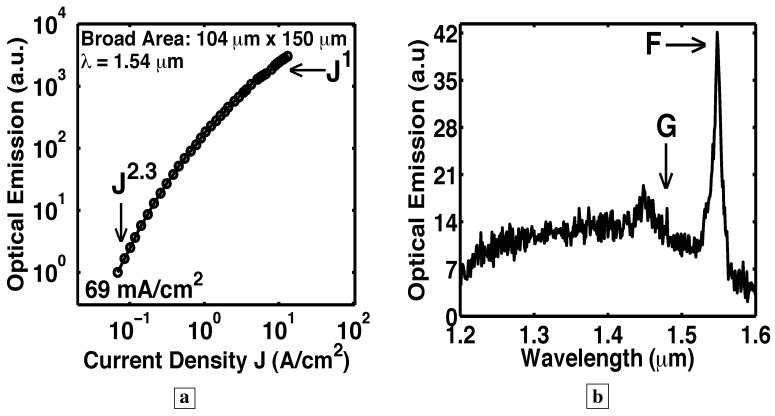
Room-temperature measurements [5] in cw-mode from a resonant cavity with both a bottom DBR and RIE facets. **a,** The stimulated-emission at 1.54 *μ*m from Sample M is seen to rise as J${}^{2.3}$, beyond which the optical-emission quickly becomes linear in J. The threshold current density is observed to be less than 69 mA/cm${}^{2}$. **b,** Optical-emission spectrum from Sample M at 7 A/cm${}^{2}$. All samples having a resonant cavity (bottom DBR plus RIE facets) showed that the low-loss total-internal-reflection mode, labeled G in Figure 18b, is suppressed, while the lossy vertical mode, labeled F in Figure 18b, dominates as the narrow spectral peak. This signifies that enough material gain exists to overcome the large 70% transmission loss incurred by the vertical mode with each trip to the sample surface.

**Figure 19:**
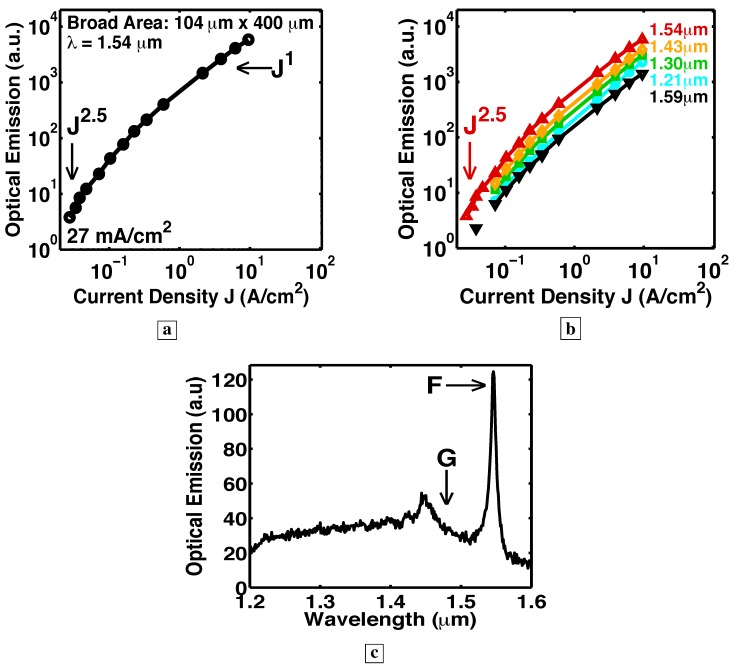
Room-temperature measurements in cw-mode from a resonant cavity. **a,** The stimulated-emission at 1.54 *μ*m from Sample N rises as J${}^{2.5}$, beyond which the optical-emission quickly becomes linear in J. The threshold current density is observed to be less than 27 mA/cm${}^{2}$. **b,** The constant vertical separation on a log-log plot of the L-I curves at different wavelengths from the same pixel indicates that the optical-emission has the same spectral shape for all indicated J, and that the carrier distribution is pinned among the energy-levels. (Without the resonant cavity, the optical emission shows a significant shift in the population distribution with increasing injection.) **c,** Optical emission spectrum from Sample N at 25 A/cm${}^{2}$. With a resonant cavity, the low-loss TIR mode, labeled G in Figure 19c, is suppressed, while the lossy vertical mode, labeled F in Figure 19c, dominates the spectra as a narrow peak. Thus, enough material gain exists to overcome the large 70% transmission loss incurred by the vertical mode with each trip to the sample surface.

Figure 14, Figure 15, Figure 16, Figure 17, Figure 18 and Figure 19 show [5] measured L-I curves and spectra at room-temperature in cw-mode. Figure 14 shows the single-pass L-I curves from the same pixel on Sample G at different wavelengths. With the longer optical-path, the stimulated-emission at 1.35 *μ*m, the longest wavelength for TIR, shows a rise as J${}^{9}$ (which is a much sharper rise than from Sample F). At J>65 A/cm${}^{2}$, the optical-emission at 1.35 *μ*m becomes proportional to the first power of J. At these J, the longer wavelength optical-emission (e.g., at 1.45 *μ*m for the lossy vertical mode of Figure 1f, and also at 1.55 *μ*m) clamps at a constant value (zero slope in the L-I curve). The latter indicates that, at J>65 A/cm${}^{2}$, all the additional carriers supply the optical emission into the low-loss TIR mode, rather than the longer wavelength lossy modes. These two observations indicate gain pinning and single-pass laser action from Sample G. Wavelengths shorter than 1.35 *μ*m also show stimulated-emission, and correspond to the modes in Figure 1h.

### 6.5. Longer optical path with a resonant cavity

In the six Samples H–N, RIE facets made possible a resonant cavity and optical-feedback. See Figure 1e. Figure 15, Figure 16, Figure 17, Figure 18 and Figure 19 show L-I curves and spectra from the six samples H–N [5]. Each pixel has been labeled by its length L, the measurement wavelength *λ*, the threshold current density (where the log-log plot of L-I has its greatest slope), and the functional form J${}^{s}$ of the stimulated-emission. With the optical-feedback resulting from RIE facets, the stimulated-emission is seen to rise even more sharply as J${}^{s}$, where *s* is as large as 27, or even 64. (See samples H and K in Figure 15a and Figure 16, respectively.) Figure 15b shows sample J to have an “S-shaped” L-I curve, which indicates a transition from spontaneous-emission, to stimulated-emission, towards laser-action. The stimulated-emission at 1.54 *μ*m from sample L is seen in Figure 17 to rise a significant three orders of magnitude as J${}^{11}$, beyond which the optical-emission quickly becomes linear in J. The latter indicates gain pinning and laser action. The threshold is less than 2 A/cm${}^{2}$. Figure 18 and Figure 19 show the stimulated-emission at 1.54 *μ*m from samples M and N to rise as J${}^{2.3}$ and J${}^{2.5}$, respectively, beyond which the optical-emission quickly becomes linear in J. The latter indicates gain pinning and laser action. The threshold is less than 69 mA/cm${}^{2}$ and 27 mA/cm${}^{2}$, respectively, for samples M and N.

### 6.6. Optical emission spectra

**Figure 20:**
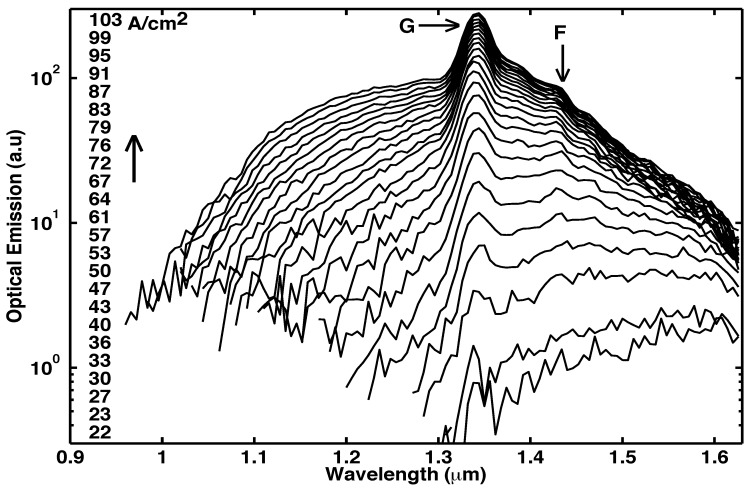
Room-temperature optical-emission spectra [5] measured from the same pixel on Sample G in a single-pass geometry at different current densities. As expected, the low-loss total-internal-reflection mode, labeled G in Figure 20, dominates the spectra as narrow peaks, while the lossy vertical mode, labeled F in Figure 20, is suppressed. (Since no top DBR has been placed on Sample G, the normal component K${}_{\text{Z}}$ of the wave vector has a broad continuum of values, as determined by the broad cavity resonance in Figure 23a below. Consequently, the Fabry-Perot modes are broadened by the width of the cavity resonance.)

Figure 20 shows the spectra from Sample G at the indicated J [5]. Figure 17c, Figure 18b, and Figure 19c show the measured room-temperature spectra [5] for samples L, M, and N. The inset in Figure 17c shows that the peak at 1.54 *μ*m is TE polarized. After correcting for the spectrometer resolution, the width of the spectral peaks in Figure 17c, Figure 18b, Figure 19c, and Figure 20 was 12 nm. (Since the vertical mode of Figure 1f is quite lossy and has a low Q, the Fabry-Perot modes in the spectra of Figure 17c, Figure 18b, Figure 19c, and Figure 20 would show significant spectral overlap, and thus could not be individually resolved.)

### 6.7. Loss in optical modes, and the observation of a gain larger than a significant loss

Another indicator of laser action is the observation of an optical gain large enough to overcome a significant loss [5]. We would expect that the spectral peaks in Figure 20, Figure 17c, Figure 18b, and Figure 19c correspond to the low-loss longest wavelength TIR mode of Figure 1g, rather than the lossy vertical mode of Figure 1f. (The labels F and G in Figure 20, Figure 17c, Figure 18b, and Figure 19c correspond to the modes shown in Figure 1f and Figure 1g, respectively.) Certainly, the single-pass measurement of Sample G in Figure 20 indeed shows its spectral peak G at the low-loss TIR mode. In conventional vertically emitting lasers, the lossy vertical mode of Figure 1f never shows laser action unless *both* a top *and* bottom DBR are present. (In conventional slab waveguide lasers, laser action does not take place unless *both* a top *and* bottom cladding layer are present, and the lossy vertical mode of Figure 1f never shows laser action.) Without a top DBR, the vertical mode of Figure 1f suffers a 70% transmission loss with every reflection at the sample surface. Thus, in the six samples H–N, it is very significant that the lossy vertical mode dominates Figure 17c, Figure 18b, and Figure 19c as the narrow spectral peak F, while the low-loss TIR mode, labeled G in Figure 17c, Figure 18b, and Figure 19c, is suppressed. The latter signifies that enough material gain exists to overcome the large 70% transmission loss incurred by the vertical mode with each trip to the sample surface. This large gain, which is needed to overcome the large loss, is another indicator of laser action. Since the vertical mode makes far more passes through the active layer than any of the TIR modes (compare Figure 1f, g, h), the lossy vertical mode acquires a higher net gain, and dominates Figure 17c, Figure 18b, and Figure 19c.

### 6.8. Estimate of the optical gain

The fact that the lossy vertical mode dominates the optical emission spectra is quite striking. The reason is that our measurement geometry (from the sample edge) was chosen to collect all emission from *TIR modes*, whose Poynting vector is parallel to the sample surface. This geometry does not collect most of the emission from the lossy vertical mode, whose Poynting vector is normal to the sample surface. Yet, the lossy vertical mode dominates the optical emission spectra even in this suboptimal measurement geometry. With a 70% transmission loss with each reflection at the sample surface and a very long optical path length, the only way that, even in this suboptimal measurement geometry, the lossy vertical mode can achieve a higher optical emission than any of the TIR modes is if net gain is achieved in one round trip (from the surface down vertically to the DBR and back up vertically to the surface). Using 210 nm for the thickness of the deep-center layer and a loss dominated by the 70% transmission, the gain for the lossy vertical mode is found to be $2.9\times 10^{4}$ cm${}^{-1}$. (The thickness of the electrically pumped portion of the deep-center layer, one hole diffusion length, is much less than 210 nm. So the gain of the lossy vertical mode may be higher than this estimate.) This is a large gain at these injection densities (27 mA/cm${}^{2}$ to 2 A/cm${}^{2}$).

Three observations explain how a large optical gain is achieved even at low injection. First, the Franck-Condon effect allows transparency to be achieved even at nearly zero injection. Second, the electrically pumped region extends only one hole diffusion length into the deep-center layer. The small volume of the electrically pumped region contains a small total number of deep-centers, and allows for a smaller threshold current. Moreover, the injected holes occupy a smaller volume, and thus achieve a higher concentration. Third, fast depopulation of the lower state of the optical transition (e.g., fast capture of free holes onto deep-centers) allows a population inversion to be easily maintained.

### 6.9. Net gain over a wide wavelength range

We have observed that the lossy vertical mode of Figure 1f (labeled F in Figure 17c, Figure 18b, and Figure 19c) has sufficient single-pass gain to overcome the 70% transmission loss with every reflection at the sample surface. This implies that shorter wavelength TIR modes (e.g., Figures 1g-h) also show net gain in a single-pass [5] because TIR modes suffer *no transmission* loss at the sample surface. Gain (and laser action) is then observed over a wide wavelength range [44]. Indeed, at every wavelength between 1.2 *μ*m and 1.6 *μ*m, the L-I curves from samples K, L, and N in Figure 16b, Figure 17b, and Figure 19b all show stimulated-emission regimes, followed by gain pinning and laser action. A consequence of the optical-resonator is thus to reduce the threshold at all wavelengths between 1.2 *μ*m and 1.6 *μ*m.

### 6.10. Carrier population pinning

Figure 8, Figure 11, Figure 13, Figure 14, and Figure 20 show that, in a single-pass through Samples F and G, the optical-emission at shorter wavelengths increases with increasing injection [5,6]. The shift to shorter wavelengths of the optical emission with increasing injection was reported in Figure 4 in [7] for a sample fabricated without a waveguide or optical resonator. For a one decade increase in the injection, from 2 A/cm${}^{2}$ to 20 A/cm${}^{2}$, the optical-emission from Sample F, which has no optical resonator, shows a dramatic shift from a peak centered at 1.58 *μ*m to a peak centered at 1.27 *μ*m. This is shown [6] in Figure 13. For a similar increase in the injection, from 2 A/cm${}^{2}$ to 17 A/cm${}^{2}$, the optical-emission from Sample L (which does have an optical resonator) shows no change in the spectral shape [5]. This is indicated by the constant vertical separation of the curves at different wavelengths from the same pixel in Figure 17b. Similarly, for a 400-fold increase in the injection, from 27 mA/cm${}^{2}$ to 10 A/cm${}^{2}$, the optical-emission from Sample N (which does have an optical resonator) shows no change in the spectral shape. This is indicated by the constant vertical separation of the curves at different wavelengths from the same pixel in Figure 19b. For a similar increase of 400-fold in the injection, Figure 13 shows the optical-emission from Sample F, which has no optical resonator, to exhibit a dramatic shift from a peak centered at 1.59 *μ*m to a peak centered at 1.1 *μ*m. This shift to shorter wavelengths of the optical emission with increasing injection indicates that the carrier population shifts significantly among the energy-levels in the absence of an optical resonator.

In contrast, when an optical resonator is added, the spectra radiated by the six samples H–N all had the same shape [5] (shown in Figure 17c, Figure 18b, and Figure 19c) at all current densities measured. (This is indicated by the constant vertical separation of the curves at different wavelengths from the same pixel in the log-log plots of Figure 16b, Figure 17b, and Figure 19b.) Thus, in the presence of an optical resonator, the carrier population has the *same distribution among the energy-levels* for all injection currents shown in Figure 16b, Figure 17b, and Figure 19b. This striking observation indicates that, even though the photon number is rising sharply, the carrier distribution among the energy-levels is pinned. Carrier population pinning is another indicator of laser action.

### 6.11. Observation of increased radiative recombination rate with a resonant cavity

The data in Figure 15, Figure 16, Figure 17, Figure 18 and Figure 19 actually show that the radiative recombination rate increases in the presence of a resonant cavity. This can be argued as follows. The hole population N${}_{\text{holes,dl}}$ in deep levels

**Figure 21:**
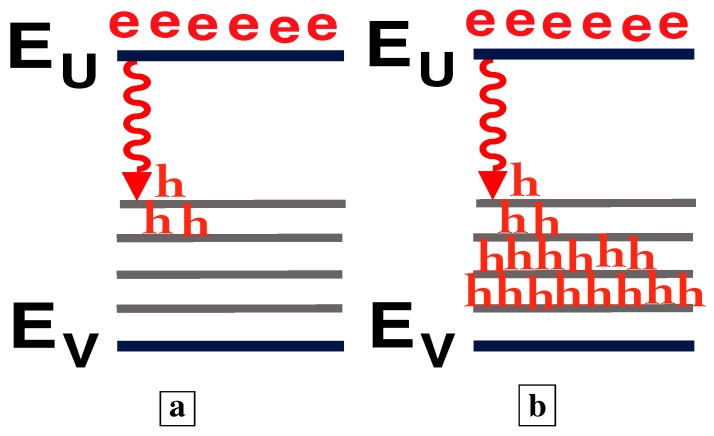
Electron and hole population distribution in Sample F (no resonant cavity). **a,** At small injection, holes occupy mainly midgap states. **b,** At higher current injection, band filling occurs. In the absence of a resonant cavity, the number of holes in deep-levels increases with increasing injection, as seen in Figure 13.

**Figure 22:**
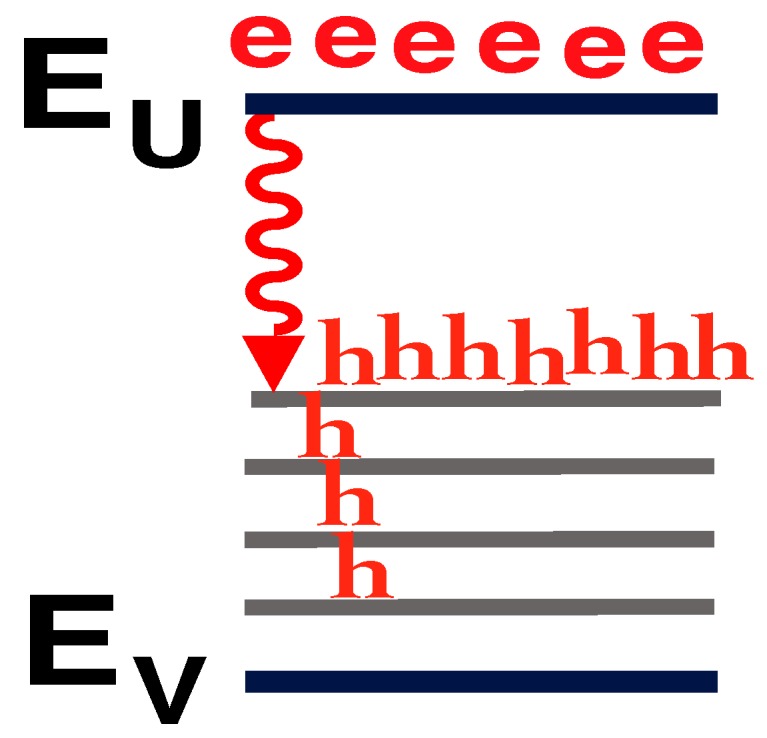
Electron and hole population distribution in Samples H–N (with resonant cavity). In the presence of a resonant cavity, Figure 15, Figure 16, Figure 17, Figure 18 and Figure 19 show that the hole population remains pinned in midgap states for all injections above threshold. The hole population in deep-levels no longer increases with increasing injection. Hence, the rate of radiative recombination of holes with electrons is greater than in Figure 21.

is related to the injected flux F${}_{\text{injected}}$ of holes per unit volume per unit time and the recombination rate R${}_{\text{eh}}$ of holes with electrons through,

(1) $$dN_{\text{holes,dl}}\;/\;dt=F_{\text{injected}}-N_{\text{holes,dl}}\;R_{\text{eh}}$$

In the steady state, the injected flux equals the recombination flux, and the steady state concentration of holes in deep-levels is,

(2) $$N_{\text{holes,dl}}=F_{\text{injected}}\;/\;R_{\text{eh}}$$

Thus, for a fixed injection, a smaller steady state concentration of holes in deep-levels implies a higher recombination rate R${}_{\text{eh}}$.

Figure 21 and Figure 22 show the electron and hole population distributions in Samples F and H–N, respectively (without and with a resonant cavity, respectively). Figure 21 shows band filling at higher injection in the absence of a resonant cavity. At higher injection, the additional holes populate states further down from the midgap and closer to the valence band. Here, in the absence of a resonant cavity, the number of holes in deep-levels increases with increasing injection. This describes the spectra shown in Figure 13. The earlier discussion of Figure 15, Figure 16, Figure 17, Figure 18 and Figure 19 shows that, in the presence of a resonant cavity, the electron and hole populations remain pinned for all current injections greater than threshold. This is shown in Figure 22. The spectra in Figure 15, Figure 16, Figure 17, Figure 18 and Figure 19 show that holes remain pinned mainly in midgap states for all injections above threshold. Thus, unlike the situation in Figure 21, the hole population in deep-levels within a resonant cavity no longer increases with increasing injection.

In fact, for a fixed injection current density, the hole concentration in the presence of a resonant cavity (Samples H–N in Figure 22) is smaller than the hole concentration in the absence of a resonant cavity (Sample F in Figure 21). For a fixed injection, a higher hole concentration is associated with Sample F in Figure 21 because higher hole concentrations are accompanied by a shift of the hole population to states closer to the valence band. Samples H–N in Figure 22 show no such shift of the hole population to states closer to the valence band. According to Equation (2), the smaller steady-state hole concentration implies a higher recombination rate R${}_{\text{eh}}$ in Samples H–N in the presence of a resonant cavity. This makes sense because the stimulated emission rate is proportional to the photon population, and the latter is greater in the presence of a resonant cavity.

### 6.12. Stimulated-emission and Einstein B coefficient

The previous section showed that, for a fixed injection, the steady-state concentration of holes is a measure of the electron-hole recombination rate: a small steady-state concentration of holes would result from a large electron-hole recombination rate. We also showed that the electron-hole recombination rate is definitively larger in the presence of a resonant cavity. Since the resonant cavity enhances the number of photons and the stimulated-emission into resonant modes, the increased electron-hole recombination is a result of an increased stimulated-emission rate. This direct observation of an increased stimulated-emission rate indicates that the radiative emission dominates in the presence of the resonant cavity. The latter indicates a high radiative efficiency. The observation of an increased stimulated-emission rate is surprising because the photon population is small: the injected current density (and the photon density) is very low, and the resonant cavity has very low Q (the transmission loss through the sample surface is 70% and the RIE etch was only 4.5 periods into the bottom DBR). The noticeably larger stimulated-emission rate, even at the low photon densities resulting from low injection in a low-Q cavity, indicates a sizable Einstein B coefficient. In a previous work [7], we estimated a sizable Einstein B coefficient of 8.2 × 10${}^{-10}$ cm${}^{3}$/s by equating the injection flux with the total electron-hole recombination flux. Although such estimates always involve a significant margin of error, they are, at least, consistent with our observation of a high stimulated-emission rate even at the low photon densities which result from a low injection density in a low-Q cavity.

Historically, it has been widely thought that point defects should show only weak radiative transitions. However, the deep-centers in this material are *not* simple point defects, but are believed to be native deep-acceptor complexes: e.g., complexes consisting of a vacancy-on-gallium-site and a donor-on-gallium-site. The radiative transition occurs between an electron on a donor-on-gallium-site and a hole on a vacancy-on-gallium-site. Since the vacancy-on-gallium-site and the donor-on-gallium-site are next-nearest neighbors, the wave function overlap and the optical dipole is significant. A similar situation occurs in F-center (i.e., color-center) lasers, wherein a strong radiative transition occurs at anion vacancies in alkali halides. A future research direction should be directed at understanding this optical transition strength.

### 6.13. Polarization of the optical emission

In the presence of the resonant cavity, the narrow spectral peak at 1.54 *μ*m in Figure 17c, Figure 18b, and Figure 19c is found to be TE-polarized [5]. This is demonstrated in the inset in Figure 17c. This is a sensible observation, because the reflectivity of TE-polarized radiation is greater than that of TM-polarized radiation for all incident angles. With the greater reflectivity at each interface, TE-polarized radiation shows better confinement in the DBR waveguide.

**Figure 23:**
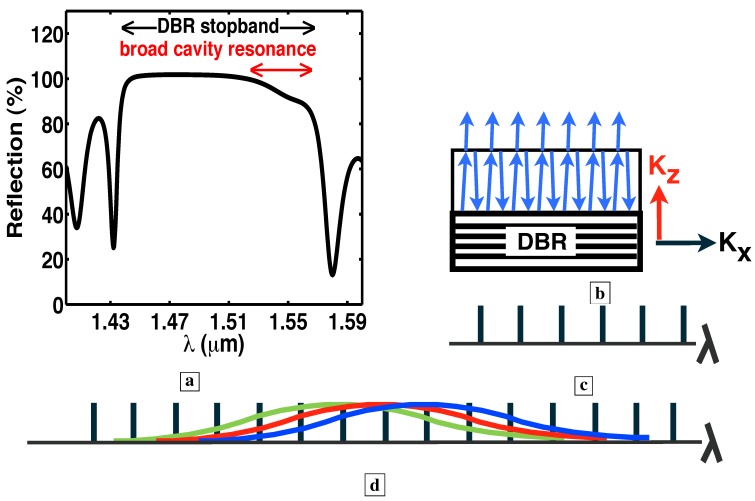
The Fabry-Perot modes of the GaAs deep-center laser. **a,** Reflection at normal incidence from the top surface of the wafer. Since the device structure has no top DBR, the normally incident optical modes show a 70% transmission loss, and the cavity resonance is broad. **b,** The lossy vertical mode (blue arrows) has a K${}_{\text{Z}}$ determined by the broad cavity resonance in Figure 23a. **c,** If K${}_{\text{Z}}$ were fixed at a single value, then the Fabry-Perot spectrum would be determined by the discrete values of the longitudinal K${}_{\text{X}}$. **d,** Since K${}_{\text{Z}}$ has a continuum of values, as determined in Figure 23a, the Fabry-Perot modes are broadened by the width of the resonance.

### 6.14. Spectral broadening of the Fabry-Perot modes

Individual Fabry-Perot modes of the GaAs deep-center laser were difficult to resolve, because the large waveguide loss broadens the spectrum of individual modes. This is shown in Figure 23. It is important to note that the device is not a vertical-cavity-surface-emitting laser (VCSEL), because the structure has no top DBR mirror. Thus, an optical mode, originating from within the semiconductor and normally incident upon the sample surface, would show a spectrally broad cavity resonance. The cavity resonance is indicated in Figure 23a as the spectrally broad dip in the reflection stopband at normal incidence. Figure 23b shows the lossy vertical mode as blue arrows. The normal component K${}_{\text{Z}}$ of the wave vector of this mode attains a broad continuum of values, as determined by the broad cavity resonance in Figure 23a. If K${}_{\text{Z}}$ were fixed at a single value, then the Fabry-Perot spectrum would be determined by the discrete values of the longitudinal component K${}_{\text{X}}$ of the wave vector, as pictured in Figure 23c. These discrete values of K${}_{\text{X}}$ are determined by boundary conditions on the electric and magnetic fields. Since the actual K${}_{\text{Z}}$ has a continuum of values, determined by the broad cavity resonance in Figure 23a, the Fabry-Perot modes are broadened by the width of the resonance, as shown in Figure 23d. The Fabry-Perot modes merge, and individual modes are difficult to resolve. Thus, since the vertical mode of Figure 1f is quite lossy and has a low Q, the Fabry-Perot modes in Figure 17c, Figure 18b, and Figure 19c would show significant spectral overlap, and thus could not be individually resolved.

### 6.15. Effect of lossy cavity on mode structure

Most laser cavities are designed to have low loss. The spectral width of these low-loss optical modes is usually much less than the spectral spacing between modes. The large loss inherent in the GaAs deep-center laser mode, shown in Figure 23, has unexpected implications for the mode structure. The broad spectral width of lossy optical modes has important implications for the number of modes which go into laser action. For example, in random lasers with non-resonant feedback [48], where the spectral broadening of each mode is greater than the spectral spacing between modes, the large number of modes within the spectral broadening are coupled together. These modes go through threshold and laser action together. Thus, even at threshold, these random lasers are not single-mode, but have a large number of coupled modes going through threshold together. The GaAs deep-center laser is exhibiting something similar. The large number of modes within the spectral width of the broad cavity resonance in Figure 23a simultaneously go through threshold and laser action together. Thus, even at low injection, the GaAs deep-center laser is not expected to be single-mode.

### 6.16. Small injection to achieve laser action

The surprisingly small injection which achieves stimulated-emission and laser action can be explained [7] by the very efficient fast capture of free holes onto deep-centers. The physics which explains the fast hole capture onto deep-centers is that, in compensated semiconductors, the deep-acceptor complexes are negatively charged, and thus exhibit a large capture cross-section for positively-charged holes. The coefficient for capture of a free hole onto the native deep-acceptors (mainly V${}_{\text{Ga}}$ and their complexes) in n-type GaAs has been measured [10] to be 2 × 10${}^{-6}$ cm${}^{3}$-s${}^{-1}$. For our deep-acceptor concentration of 2 × 10${}^{19}$ cm${}^{-3}$, this coefficient yields a capture lifetime for free holes of about 25 fs, and corresponds to a free hole diffusion length of 25 Å. Recent pump-probe measurements [32,33] have determined the capture lifetime of a free hole onto V${}_{\text{Ga}}$ to be 100 fs. This agrees with our previous estimate [7] based upon determining a free hole diffusion length L${}_{P}$ of 100 Å in the deep-center material. This hole diffusion length is thus 10${}^{4}$ times smaller than in high quality GaAs [49]. The hole diffusion length is indicated schematically in the device layer structure of Figure 13a as L${}_{P}$. The latter is important for two reasons. First, when L${}_{P}$ is smaller, then holes are injected into a smaller region (one L${}_{P}$ away from the p-n junction), and thus achieve a higher concentration within the smaller region. Second, when L${}_{P}$ is smaller, then electrical injection probes a smaller total number of deep-centers: only those deep-centers within one L${}_{P}$ of the p-n junction. A smaller total number of deep-centers constitutes a smaller population which is to be inverted. Population inversion is then easier to achieve within a smaller L${}_{P}$ within the n-type deep-center layer because of: first, the higher hole concentration, and, second, the smaller total number of deep-centers, whose population is to be inverted. (In regions of the n-type deep-center layer which are more than one L${}_{P}$ away from the p-n junction, the population is not inverted, but the absorption [7,8] is very small at the wavelengths of the deep-center transitions.)

Thus, the small injection which achieves laser action is a consequence of the fast depopulation of the lower state of the optical transition (i.e., fast capture of free holes onto deep-centers). The latter is important for maintaining the population inversion. Moreover, a Franck-Condon effect allows transparency to be achieved at nearly zero injection. Finally, a large stimulated-emission rate (Einstein B coefficient) is observed even at the low photon densities which result from a low injection density in a low-Q cavity.

### 6.17. Summary

Thus far, we have demonstrated the first GaAs deep-center laser. Electrically-pumped broad-area lasers exhibited a threshold of less than 27 mA/cm${}^{2}$ in cw-mode at room-temperature at the 1.54 *μ*m wavelength. In Sample G having wet-etched facets, the longest wavelength TIR mode (Figure 1g) shows a superlinear L-I curve at low injection, and a linear regime in the L-I curve at higher injection. At injections high enough for laser action in this TIR mode, the longer wavelength optical emission is clamped at a constant value. The latter indicates that, at these injection levels, all the additional carriers supply the optical emission into the low-loss TIR mode, rather than the longer wavelength lossy modes. Both observations indicate single-pass laser action and gain pinning. With a resonant cavity and RIE facets, the stimulated-emission from sample L rises a significant three orders of magnitude as J${}^{11}$ with a threshold less than 2 A/cm${}^{2}$. The threshold is less than 69 mA/cm${}^{2}$ and 27 mA/cm${}^{2}$, respectively, for samples M and N. With a resonant cavity and RIE facets, samples H-N all show a pinning of the carrier distribution among the energy-levels at all injections greater than threshold. (Without a resonant cavity, previous work [6,7] showed that an increasing injection results in a marked shift in the carrier distribution, and a rise in shorter wavelength emission.) With a resonant cavity and RIE facets, the dominant mode in the optical emission spectrum (Figure 17c, Figure 18b, and Figure 19c) is the lossy vertical mode of Figure 1f. In order to be dominant, this lossy mode must undergo an optical gain which is large enough to overcome a significant loss. A large optical gain is another indicator of laser action.

## 7. Conclusions

We have reviewed recent work which allowed the first demonstration of a GaAs deep-center laser. First, we summarized some well-known properties of deep-centers in highly n-doped GaAs: the nature of the deep-acceptor complexes, the Franck-Condon effect, the observed photoluminescence. Second, we describe our recent work: the total radiative output in photoluminescence, the insensitivity of the photoluminescence with respect to a 90 ${}^{\circ }$C rise above room temperature, the dependence of the photoluminescence and electroluminescence on the pump power, striking differences between electroluminescence and photoluminescence, a correlation between transitions to deep-states and the absence of bandgap emission, the fast capture of free holes onto deep-centers. We observed room-temperature stimulated-emission from GaAs deep-centers at low electrical injection. The evidence for stimulated-emission included: a superlinear L-I curve, a quasi-Fermi level separation large enough to satisfy the Bernard-Duraffourg criterion, and an optical gain large enough to overcome significant loss. The room-temperature stimulated-emission from GaAs deep-centers can be tuned very widely from the bandgap (about 900 nm) to half-the-bandgap (1,600 nm) by changing the electrical injection. The first GaAs deep-center laser was demonstrated with electrical injection, and exhibited a threshold of less than 27 mA/cm${}^{2}$ in continuous-wave mode at room temperature at the important 1.54 *μ*m fiber-optic wavelength. This small injection which achieves laser action can be explained by a fast depopulation of the lower state of the optical transition (i.e., fast capture of free holes onto deep-centers). The latter helps to maintain the population inversion. The evidence for laser action included: a superlinear L-I curve, an optical gain large enough to overcome significant loss, a clamping of the optical emission from lossy modes that do not show laser action, and a pinning of the population distribution during laser action.

## 8. Outlook

Three obvious directions of future research are: very low threshold lasers, tunability over a wide spectral range, and very short pulse generation. In this review, stimulated emission and laser action were observed at very low current density. This was in spite of the fact that the optical cavities showed very significant loss (70% transmission loss through the sample surface, and only a shallow RIE etch of 4.5 periods into the bottom DBR). A higher Q cavity would result in further reduction of the operating current. The addition of a top DBR or a top cladding layer in a slab waveguide are obvious paths for increasing the Q of the cavity. We also showed that the stimulated emission is not pinned to the bandgap energy, but could be tuned from 900-1700 nm. This should allow for semiconductor lasers having a very wide spectral tuning range. A wide spectral tuning range is useful for spectroscopy, lab-on-a-chip, chemical species identification, fiber-optics, and medicine. Optical gain spanning 1.3 to 1.6 *μ*m in the same semiconductor is very unique, and could prove useful for very dense wavelength division multiplexing. Such tunable semiconductor lasers would be compatible with integration on GaAs and mass production. A further application for GaAs deep-centers is the generation of pulses having a very short duration. The latter requires optical gain over a wide spectral range, such as was observed in this review.

## Supplementary Materials

Supplementary File 1

## References

[1] Park H.G., Kim S.H., Kwon S.H., Ju Y.G., Yang J.K., Baek J.H., Kim S.B., Lee Y.H. (2004). Electrically driven single-cell photonic crystal laser. Science.

[2] Agrawal G.P., Dutta N.K. (1986). Long-Wavelength Semiconductor Lasers.

[3] Reitzenstein S., Bazhenov A., Gorbunov A., Hofmann C., Muench S., Loeffler A., Kamp M., Reithmaier J.P., Kulakovskii V.D., Forchel A. (2006). Lasing in high-Q quantum-dot micropillar cavities. Appl. Phys. Lett..

[4] Luo K.J., Xu J.Y., Cao H., Ma Y., Chang S.H., Ho S.T., Solomon G.S. (2001). Ultrafast dynamics of InAs/GaAs quantum-dot microdisk lasers. Appl. Phys. Lett..

[5] Gupta M., Pan J.L. (2009). Gallium-arsenide deep-center laser. Appl. Phys. B: Lasers Opt..

[6] Gupta M., Pan J.L. (2009). Stimulated-emission from GaAs deep-centers. Opt. Exp..

[7] Pan J.L., McManis J.E., Gupta M., Young M.P., Woodall J.M. (2008). Novel deep-centers for high-performance optical-materials. Appl. Phys. A.

[8] Williams E.W. (1968). Evidence for self-activated Luminescence in GaAs: The gallium vacancy-donor center. Phys. Rev..

[9] Vorobkalo F.M., Glinchuk K.D., Prokhorovich A.V., John G. (1973). Effect of heat-treatment on 0.93, 1.0, and 1.28eV luminescence bands in n-GaAs. Phys. Stat. Sol. (a).

[10] Vorobkalo F.M., Glinchuk K.D., Prokhorovich A.V. (1971). Characteristics of 0.93 to 1.0eV luminescence bands in GaAs. Phys. Stat. Sol. (a).

[11] Lei H., Leipner H.S., Bondarenko V., Schreiber J. (2004). Identification of the 0.95 eV luminescence band in n-type GaAs:Si. J. Phys.: Cond. Matt..

[12] Tajima M., Toba R., Ishida N., Warashina M. (1997). Optical and electrical non-uniformity around dislocations in silicon doped GaAs. Mat. Sci. Tech..

[13] Reshchikov M.A., Gutkin A.A., Sedov V.E. (1995). Structure of the 0.95eV photoluminescence centers in n-type GaAs. Mat. Sci. Forum.

[14] Suezawa M., Kasuya A., Nishina Y., Sumino K. (1991). Optical studies of heat-treated Si-doped GaAs bulk crystals. J. Appl. Phys..

[15] Kung J.K., Spitzer W.G. (1974). Si-defect concentrations in heavily Si-doped GaAs: Changes induced by annealing. J. Appl. Phys..

[16] Chiang S.Y., Pearson G.L. (1975). Photoluminescence studies of vacancies and vacancy-impurity complexes in anealed GaAs. J. Luminescence.

[17] Sauncy T., Palsule C.P., Holtz M., Gangopadhyay S., Massie S. (1996). Lifetime studies of self-activated photoluminescence in heavily silicon-doped GaAs. Phys. Rev. B.

[18] Suezawa M., Kasuya A., Nishina Y., Sumino K. (1994). Excitation spectra of 1200 and 1320 nm photoluminescence lines in annealed gallium arsenide doped with silicon. J. Appl. Phys..

[19] Ebert Ph. (2001). Atomic structure of point defects in compound semiconductor surfaces. Curr. Opin. Solid St. M..

[20] Gebauer J., Lausmann M., Staab T.E.M., Krause-Rehberg R., Hakala M., Puska M.J. (1999). Microscopic identification of native donor Ga-vacancy complexes in Te-doped GaAs. Phys. Rev. B.

[21] Domke C., Ebert Ph., Urban K. (1998). Changes of defect and active-dopant concentrations induced by annealing of highly Si-doped GaAs. Phys. Rev. B.

[22] Domke C., Ebert Ph., Heinrich M., Urban K. (1996). Microscopic identification of the compensation mechanisms in Si-doped GaAs. Phys. Rev. B.

[23] Gebauer J., Krause-Rehberg R., Domke C., Ebert Ph., Urban K. (1997). Identification and quantification of defects in highly Si-doped GaAs by positron annihilation and scanning tunneling microscopy. Phys. Rev. Lett..

[24] Ito H., Furuta T., Ishibashi T. (1991). Minority electron lifetimes in heavily doped p-type GaAs grown by molecular beam epitaxy. Appl. Phys. Lett..

[25] Casey H.C., Stern F. (1976). Concentration-dependent absorption and spontaneous emission of heavily doped GaAs. J. Appl. Phys..

[26] Nelson R.J., Sobers R.G. (1978). Minority-carrier lifetimes and internal quantum efficiency of surface-free GaAs. J. Appl. Phys..

[27] Maassdorf A., Gramlich S., Richter E., Brunner F., Weyers M., Traenkle G., Tomm J.W., Mazur Y.I., Nickel D., Malyarchuk V., Guenther T., Lienau Ch., Baerwolff A., Elsaesserm T. (2002). Minority-carrier kinetics in heavily doped GaAs:C studied by transient photoluminescence. J. Appl. Phys..

[28] Yablonovitch E., Cody G. (1982). Intensity enhancement in textured optical sheets for solar cells. IEEE Trans. Electron Devices.

[29] Yablonovitch E. (1982). Statistical ray optics. J. Opt. Soc. Amer..

[30] Pan J.L. Surface and interface studies of GaAs deep-centers for high-efficiency 1.3um-1.5um fiber-optic light-emitters. Proceedings of the 34th Conference on the Physics and Chemistry of Semiconductor Interfaces (PCSI-34).

[31] Pan J.L. Fast carrier dynamics in GaAs deep-centers for novel high-efficiency light-emitters for 1.3um-1.5um fiber optics. SPIE Proceedings, Photonics West, Optoelectronics 2007 Symposium on Integrated Optoelectronic Devices, Conference 6471A: Ultrafast Phenomena in Semiconductors and Nanostructure Materials XI.

[32] Melloch M.R., Woodall J.M., Harmon E.S., Otsuka N., Pollak F.H., Nolte D.D., Feenstra R.M., Lutz M.A. (1995). Low-temperature grown III-V materials. Ann. Rev. Mater. Sci..

[33] Melloch M.R., Nolte D.D., Woodall J.M., Chang J.C.P., Harmon E.S. (1996). Molecular beam epitaxy of nonstoichiometric semiconductors and multiphase material systems. Crit. Rev. Sol. St. Mater. Sci..

[34] Siegman A.E. (1986). Lasers.

[35] Yariv A. (1975). Quantum Electronics.

[36] Yamamoto Y., Slusher R.E. (1993). Optical processes in microcavities. Phys. Today.

[37] Rex N.B., Chang R.K., Guido L.J. (2001). Threshold lowering in GaN micropillar lasers by means of spatially selective optical pumping. IEEE Phot. Tech. Lett..

[38] Chang S., Rex N.B., Chang R.K., Chong G., Guido L.J. (1999). Stimulated emission and lasing in whispering-gallery modes of GaN microdisk cavities. Appl. Phys. Lett..

[39] Ates S., Ulrich S.M., Michler P., Reitzenstein S., Loeffler A., Forchel A. (2007). Coherence properties of high-*β* elliptical semiconductor micropillar lasers. Appl. Phys. Lett..

[40] Hall R.N., Fenner G.E., Kingsley J.D., Soltys T.J., Carlson R.O. (1962). Coherent light emission from GaAs junctions. Phys. Rev. Lett..

[41] Khan M.A., Olson D.T., Van Hove J.M., Kuznia J.N. (1991). Vertical-cavity, room-temperature stimulated emission from photopumped GaN films deposited over sapphire substrates using low-pressure metalorganic chemical vapor deposition. Appl. Phys. Lett..

[42] Jacobs R.R., Samelson H., Lempicki A. (1973). Losses in cw dye lasers. J. Appl. Phys..

[43] Bernard M.G.A., Duraffourg B. (1961). Laser conditions in semiconductors. Phys. Stat. Sol..

[B44-materials-02-01599] 44.Inhomogeneously broadened quantum dot transitions, which show photoluminescence over a 82 nm FWHM, will then exhibit laser action over a broad 40 nm wavelength range [45,46,47]. The literature [45,46,47] shows that when the homogeneous broadening is less than the inhomogeneous broadening, the ensemble of quantum-dots acts as an array of independent lasers. In such cases, individual Fabry-Perot modes cannot be observed.

[45] Djie H.S., Ooi B.S., Fang X.M., Wu Y., Fastenau J.M., Liu W.K. (2007). Room-temperature broadband emission of an InGaAs / GaAs quantum dots laser. Opt. Lett..

[46] Tan C.L., Wang Y., Djie H.S., Ooi B.S. (2007). Role of optical gain broadening in the broadband semiconductor quantum-dot laser. Appl. Phys. Lett..

[47] Markus A., Chen J.X., Paranthoen C., Fiore A., Platz C., Gauthier-Lafaye O. (2003). Simultaneous two-state lasing in quantum-dot lasers. Appl. Phys. Lett..

[48] Cao H., Ling Y., Xu J.Y., Cao C.Q., Kumar P. (2001). Photon statistics of random lasers with resonant feedback. Phys. Rev. Lett..

[49] Sze S. (1981). Physics of Semiconductor Devices.

