# Correction: Pan, J.L. Progress to a Gallium-Arsenide Deep-Center Laser. *Materials* 2009, *2*, 1599-1635

**DOI:** 10.3390/ma2041795

**Published:** 2009-11-05

**Authors:** Janet L. Pan

**Affiliations:** Yale University, P.O. Box 208284, New Haven, CT 06520-8284, USA; E-Mail: janet.pan@yale.edu; Tel.: 203-432-4733; Fax: 203-432-6420

The author acknowledges that her former graduate students, J. E. McManis and M. Gupta, collected the data in the recent review [1], as indicated by the references therein. 

Figure 19 in Ref. [1] is unpublished data courtesy of M. Gupta and J. L. Pan.
