# Supplementary material for: Progress to a Gallium-Arsenide Deep-Center Laser
Source: Materials (Basel). 2009 Oct 22;2(4):1599–635. doi: 10.3390/ma2041599 (PMC5513387; doi:10.3390/ma2041599)
Supplement: Supplementary File 1 [file materials-02-01599-s001.pdf]

Materials **2009**, *2*, 1795; doi:10.3390/ma2041795

OPEN ACCESS

materials

ISSN 1996-1944

www.mdpi.com/journal/materials

Correction

Correction: Pan, J.L. Progress to a Gallium-Arsenide Deep-Center Laser. *Materials* 2009, *2*, 1599-1635

Janet L. Pan

Yale University, P.O. Box 208284, New Haven, CT 06520-8284, USA; E-Mail: janet.pan@yale.edu; Tel.: 203-432-4733; Fax: 203-432-6420

Received: 4 November 2009 / Published: 5 November 2009

The author acknowledges that her former graduate students, J. E. McManis and M. Gupta, collected the data in the recent review [1], as indicated by the references therein.

Figure 19 in Ref. [1] is unpublished data courtesy of M. Gupta and J. L. Pan.

References

1. Pan, J.L. Progress to a Gallium-Arsenide Deep-Center Laser. *Materials* **2009**, *2*, 1599-1635.

© 2009 by the authors; licensee Molecular Diversity Preservation International, Basel, Switzerland. This article is an open-access article distributed under the terms and conditions of the Creative Commons Attribution license (http://creativecommons.org/licenses/by/3.0/).
